# Non-coding regions of nuclear-DNA-encoded mitochondrial genes and intergenic sequences are targeted by autoantibodies in breast cancer

**DOI:** 10.3389/fgene.2022.970619

**Published:** 2023-03-29

**Authors:** Deya Obaidat, Roberta Giordo, Erica L. Kleinbrink, Emilia Banisad, Lawrence I. Grossman, Rooshan Arshad, Azadeh Stark, Marie-Claire Maroun, Leonard Lipovich, Félix Fernandez-Madrid

**Affiliations:** ^1^ Department of Internal Medicine, Division of Rheumatology, Wayne State University School of Medicine, Detroit, MI, United States; ^2^ Department of Basic Medical Sciences, College of Medicine, Mohammed Bin Rashid University of Medicine and Health Sciences, Dubai, United Arab Emirates; ^3^ Center for Molecular Medicine and Genetics, Wayne State University, Detroit, MI, United States; ^4^ Quantitative Life Sciences, McGill University, Montreal, QC, Canada; ^5^ Department of Pathology, Henry Ford Health System, Detroit, MI, United States; ^6^ Shenzhen Huayuan Biotechnology Co. Ltd, Shenzhen Huayuan Biological Science Research Institute, Shenzhen, Guangdong, China; ^7^ Karmanos Cancer Institute, Wayne State University, Detroit, MI, United States

**Keywords:** breast cancer, mitochondria, autoimmunity, long non-coding RNA, carcinogenesis, phage display

## Abstract

Autoantibodies against mitochondrial-derived antigens play a key role in chronic tissue inflammation in autoimmune disorders and cancers. Here, we identify autoreactive nuclear genomic DNA (nDNA)-encoded mitochondrial gene products (*GAPDH, PKM2, GSTP1, SPATA5, MFF, TSPOAP1, PHB2, COA4, and HAGH*) recognized by breast cancer (BC) patients’ sera as nonself, supporting a direct relationship of mitochondrial autoimmunity to breast carcinogenesis. Autoreactivity of multiple nDNA-encoded mitochondrial gene products was mapped to protein-coding regions, 3’ untranslated regions (UTRs), as well as introns. In addition, autoantibodies in BC sera targeted intergenic sequences that may be parts of long non-coding RNA (lncRNA) genes, including *LINC02381* and other putative lncRNA neighbors of the protein-coding genes *ERCC4, CXCL13, SOX3, PCDH1, EDDM3B,* and *GRB2*. Increasing evidence indicates that lncRNAs play a key role in carcinogenesis. Consistent with this, our findings suggest that lncRNAs, as well as mRNAs of nDNA-encoded mitochondrial genes, mechanistically contribute to BC progression. This work supports a new paradigm of breast carcinogenesis based on a globally dysfunctional genome with altered function of multiple mitochondrial and non-mitochondrial oncogenic pathways caused by the effects of autoreactivity-induced dysregulation of multiple genes and their products. This autoimmunity-based model of carcinogenesis will open novel avenues for BC treatment.

## Introduction

A comprehensive immunofluorescence (IFA) survey of autoantibodies in women with pathology-verified BC and benign breast disease (BBD) detected the presence of high-titer autoantibodies consistently targeting mitochondria, centrosomes, centromeres, and other organelles as well as subcellular structures (Fernández Madrid et al., 2015). These autoantibodies were also detected in the sera of a small group of healthy women with suspicious mammography assessment and BBD, suggesting that the process triggering autoantibody formation begins in the pre-malignant phase (Fernández Madrid et al., 2015) or occurs—to some extent—in the absence of malignancy. This study supports our proposal that the autoantibody profile in BC, similarly to the accepted role of autoantibodies in the pathogenesis of rheumatic autoimmune diseases (RADs) (Tan, 1982; Suurmond and Diamond, 2015), mechanistically participates in the process of carcinogenesis. Until the report that anti-mitochondrial antibodies (AMAs) are found consistently in BC sera (Fernández Madrid et al., 2015), only two diseases, primary biliary cholangitis (PBC) and pemphigus vulgaris (PV), were known to consistently exhibit AMAs in sera (Gershwin et al., 1987; Fritzler and Manns, 2002; Marchenko et al., 2010). The role of mitochondrial autoimmunity in carcinogenesis (Fernández Madrid et al., 2015) is further supported by the report that key enzyme components of the mitochondrial electron transport chain (ETC) are BC autoantigens (Maroun et al., 2017), and more recently by the demonstration that autoimmunity is responsible for the direct participation of MNRR1 in breast carcinogenesis (Aras et al., 2019). These foundational studies suggested that a comprehensive survey of mitochondrial autoreactivity in BC would provide insight into the process of carcinogenesis.

The post-genomic era has enhanced our understanding of transcriptional and post-transcriptional events, including gene regulatory processes. In addition to the canonical protein-encoding open reading frames (ORFs) of protein-coding genes, long non-coding RNAs (lncRNAs) are increasingly recognized as important players in gene regulation (Kung et al., 2013), and they occasionally encode novel short peptides translated from their internal small ORFs (smORFs) (Dragomir et al., 2020). Additionally, large numbers of short non-canonical ORFs have been discovered in the 5′-UTRs (upstream ORFs (uORFs) and 3′-UTRs (downstream ORFs (dORFs) of protein-coding genes (Barbosa et al., 2013), and it is reasonable to posit that peptides translated from lncRNA smORFs as well as from mRNA uORFs and dORFs may be antigenic. Although uORFs appear to regulate gene expression, often repressing the downstream canonical ORF of the gene’s known protein product, the function of dORFs remains unclear. A recent report suggested a translational stimulatory function of dORFs in eukaryotic genes (Wu et al., 2020). In the present study, we report the discovery and initial functional characterization of a group of autoreactive nDNA-encoded mitochondrial gene products revealed by immune-screening two complementary-DNA (cDNA) libraries, followed by microarray analyses of potential BC autoantigens that reflect the participation of mitochondrial autoimmunity in breast carcinogenesis. As evidence of the complexity of carcinogenesis, we also report a group of non-mitochondrial autoreactive lncRNAs, which may mechanistically contribute to BC progression.

## Materials and methods

### Patient cohort and sampling

From a cohort of women ≥40 years of age undergoing annual screening mammography with Breast Imaging Reporting and Data System 4 (BI-RADS4) assessment at the Henry Ford Health System (HFHS), Detroit, Michigan, United States, we selected sera from 100 women who were diagnosed with infiltrating ductal carcinoma (IDC) by pathologic exam (the sera were obtained prior to any treatment). The demographics of the study group were 60% White Americans (including of Hispanic heritage), 30% African Americans and 10% Asian Americans, Native Americans, Native Alaskan and mixed-race. Sera used as controls were obtained from 100 healthy women, selected out of the same patient population, with biopsy-proven BBD and no cancer. The demographic characteristics of these cases and controls have been previously reported (Fernández Madrid et al., 2015). Each of these women was invited to donate a 10 ml blood sample after signing an informed consent. The study was approved by the HFHS and the Wayne State University IRBs.

### Construction of complementary-DNA expression libraries

The construction and immune-screening of cDNA expression libraries, the assembly of micro-collections of the cloned phages on derivatized glass slides, the identification of unique clones, the cDNA sequence determination, and the homology searches of informative phages in databases have all been reported (Fernández Madrid et al., 2004; Maroun et al., 2017; Aras et al., 2019), and were adapted to identify the autoreactome in BC with emphasis on the mitochondria. Because immunoglobulins could paradoxically be expressed in epithelial cancer cells, and contribute to immunoglobulin synthesis, and perhaps to antigen presentation (Cui et al., 2021), we utilized two different human cDNA expression libraries to characterize the BC autoreactome: a random-primed library established BC cell lines, and a tumor tissue-derived cDNA library of commercial origin (Novagen) that we used in our previous work (Fernández Madrid et al., 2004). Our goal was to identify the source of the potential BC antigens, and to ascertain which autoantigens are generated specifically within epithelial cancer cells, without the direct contribution of any other cells present in the tumor microenvironment such as antigen-presenting cells (multi-cell line library), and those which are generated in the patient tumor tissues (tumor tissue-derived library, Novagen). The known heterogeneity of BC (Miller et al., 1988) was an additional factor in support of including the multi-BC-cell-line cDNA library. Since the inherent heterogeneity of BC in the multi-cell line library likely exceeds that in the patient-derived library, we expected to detect signals in the cell line library that are not present in the patient derived library. The multi-cell-line library was constructed by directional cloning of randomly primed cDNA from 7 human breast carcinoma cell lines (SUM 44, SUM 102, SUM 149, SUM 159, MCF7, SKBR, T47D), and a pre-malignant cell line (MCF10A), into a T7 phage display vector, using the T7 Select 10-3b vector and the Orient Express cDNA library construction system (Novagen, Billerica, MA, United States), as reported (Maroun et al., 2017). Further information about culture of BC cells and RNA extraction and purification of the multi BC cell line cDNA library construction can be found in Supplementary Material. Having a multi-cell line library in this study represents the heterogeneity of breast cancer and provides wider diversity of potential antigens than cDNA libraries of commercial origin that utilize RNA from a single case of BC. This cDNA library is novel and to our knowledge has not been reported before.

### Selection of anti-mitochondrial antibodies-enriched breast cancer sera for immune-screening complementary-DNA libraries

The approach used to facilitate the identification of potential mitochondrial autoantigens consisted of using BC sera containing high-titer AMAs (≥1:320–640 dilution), detected by IFA on human Epithelial type 2 (HEp-2) cells, as biopanning sera. A/G agarose beads (Santa Cruz Biotechnology, Santa Cruz, CA), incubated overnight with 100 μl of a 1:50 dilution of cloning sera at 4°C, were used to react with a culture of *Escherichia coli* strain BLT5403, which was then lysed and centrifuged. Up to 5 rounds of biopanning were performed on the resulting supernatant as we described. The initial rounds did not yield individual unique phages but a mixture of phages with different inserts. Hence, biopanning was repeated until lysed clones yielded unique phages, as indicated by single DNA bands by PCR.

### Autoantigen microarray development

A microarray was constructed in triplicate on fluorescent array surface technology glass slides using a Flexys robot (Genomic Systems) as reported (Fernández Madrid et al., 2004; Maroun et al., 2017; Aras et al., 2019). Briefly, plaque-pure phages, which had a single band by PCR, were grown to high titer in bacterial cultures that were incubated until complete lysis. Supernatants were collected after a 10 min, 10,000 × *g* spin and then were arrayed in 384-well microtiter dishes. Each slide printed with phage clones was hybridized either with an individual serum from the collection of patients with IDC of the breast or with a non-cancer control serum. Slides were then treated with a mouse monoclonal antibody against the non-variable T7 phage coat protein (Novagen). CY3-labeled anti-human secondary antibodies and CY5-labeled anti-mouse antibodies were used to assess patient autoreactivity and to quantify phage concentration as reported (Fernández Madrid et al., 2004). The insert size of individual clones of the complete library was analyzed by PCR as reported (Fernández Madrid et al., 2004). Biopanning with BC sera containing high-titer AMAs was followed by microarray assembly of the whole collection of identified clones, hybridization of the potential BC autoantigens with sera from cases and non-cancer controls with exclusion of sera used for biopanning, and development of the autoantigen microarray as reported (Maroun et al., 2017). Briefly, the array background intensity was subtracted from the foreground intensity. Negative values were replaced by a value equal to half the minimum positive value. The within-array median normalization was used on the log2 ratios of red and green intensities. Replicated spots for the same marker were then summarized using median intensities. The statistical significance of the reactivity of the AMAs with BC diagnosis was determined individually as reported (Aras et al., 2019). Student’s *t*-test was used for identifying the markers that differentiate IDC from the control group. PCR, sequence analyses, homology searches, hybridization of the printed phages on glass slides, and development of the autoantigen microarray and data pre-processing were performed as previously described (Fernández Madrid et al., 2004; Maroun et al., 2017).

### Sequence analysis and bioinformatics

After identifying informative phages, the corresponding cDNA inserts were amplified by PCR with primers flanking the insertion site and sequenced commercially using Sanger sequencing (Genewiz, South Plainfield, NJ). The sequences deduced from the cloned cDNA were searched for vector contamination with the NCBI VecScreen tool, and the vector-containing clones were eliminated. The remaining sequences were screened by RepeatMasker (www.RepeatMasker.org) to detect repetitive sequences. The GenBank database was searched for sequence homology to the identified cDNA sequences using the Basic Local Alignment Search Tool (BLAST) program (Altschul et al., 1994). When BLASTn showed highly significant homology, usually in the range of 98%–100% identity, to a sequence other than a protein-coding exon, we analyzed the sequence by the UCSC Genome Browser’s BLAST-like alignment tool (BLAT). The ORF-encoded proteins and the intergenic-mapping phage sequences significantly recognized by BC sera on the microarray, but not by control sera, were analyzed by BLAT. We searched for the closest coding gene to these sequences; if the closest gene was a lncRNA sequence we also searched for the closest protein-coding gene within 500 kb distance because of the well-established knowledge that non-coding RNA genes can regulate the function and expression of their neighboring protein-coding genes (Katayama et al., 2005). Homologies detected by BLASTn or BLASTx were used, the smallest E-values were recorded, and conserved domains in the query sequences were determined. All the results from homology searches were genomically mapped by the BLAT functionality of the UCSC Genome Browser (Kent et al., 2002). We used the UCSC Genome Browser (Kent et al., 2002) to manually annotate the genomic mapping locations of all autoreactive phage sequences. We used the hg19 human genome assembly (except for the phage sequence of the non-coding neighbor of *EDDM3B*, which fails to map by BLAT to hg19 and only BLAT-maps to hg38) (Supplementary Material), incorporating all pertinent browser tracks including but not limited to mRNAs, ESTs, evolutionary conservation, RNA-seq, ENCODE Consortium epigenetics data, and reference gene models from NCBI RefSeq and the Gencode human gene catalog, into the analysis. The gene ontology (GO) analysis for the whole collection of potential breast autoantigens has been partially reported (Maroun et al., 2017). BLAST and BLAT (Altschul et al., 1994; Kent et al., 2002) output from the autoantigen microarray analysis was used to generate a gene list utilized as input for GO analysis using R. The R package Biomart is a collection of databases implementing the Biomart software suite (http://www.biomart.org). It facilitates the retrieval of large amounts of data with a simple query (Bio-Conductor version: Release 3.2). The analysis created a text file of GO identifiers corresponding to the BLAST/BLAT input gene list.

## Results

### Nuclear genomic DNA-encoded mitochondrial gene products are targeted by autoantibodies in breast cancer sera

**FIGURE 1:**
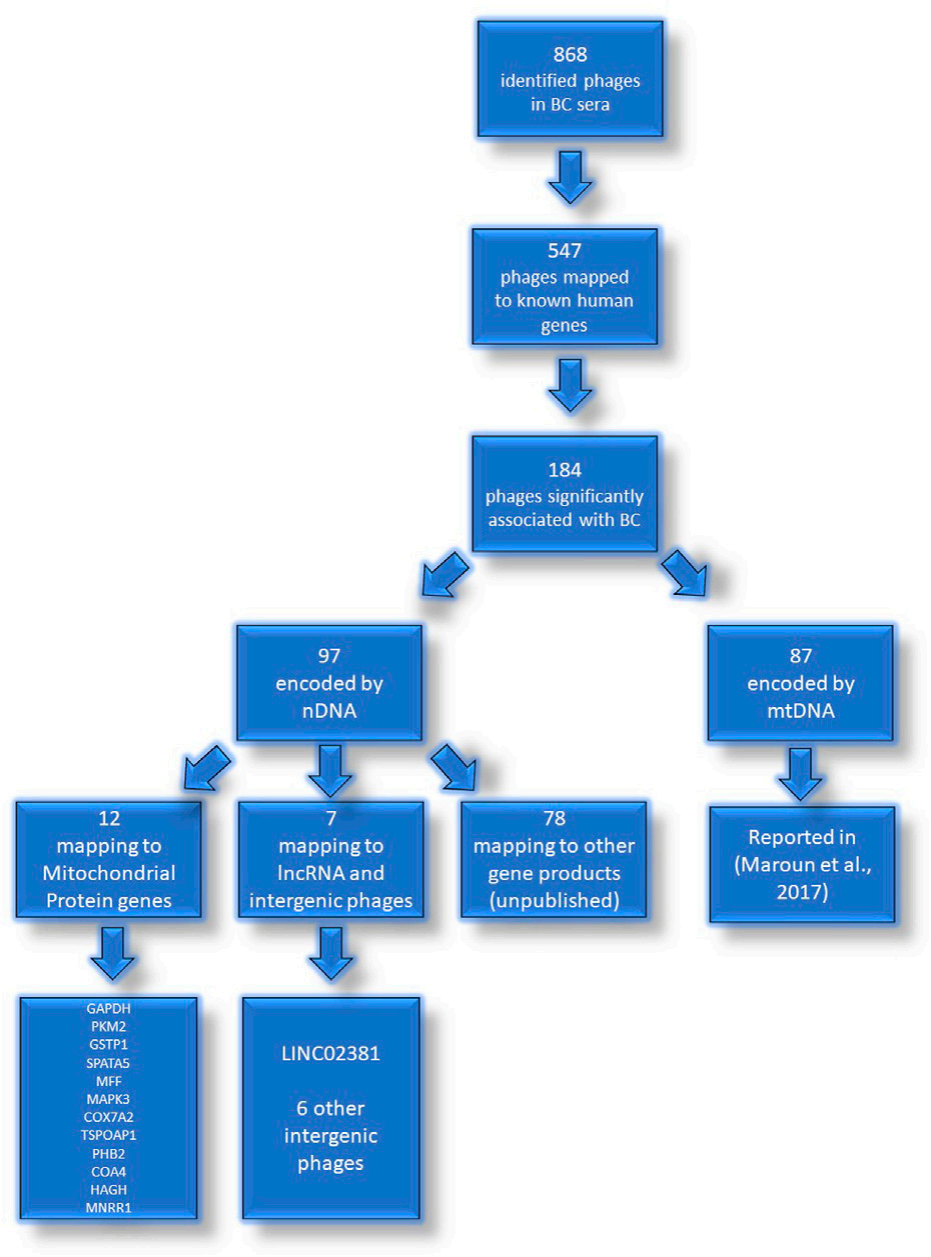
Identified phages in sera of patients with BC. Flowchart representation of the experimental identification of autoantibody targets in BC patients. The mtDNA genes targeted by autoantibodies in BC were reported previously (Maroun et al., 2017).

**FIGURE 2:**
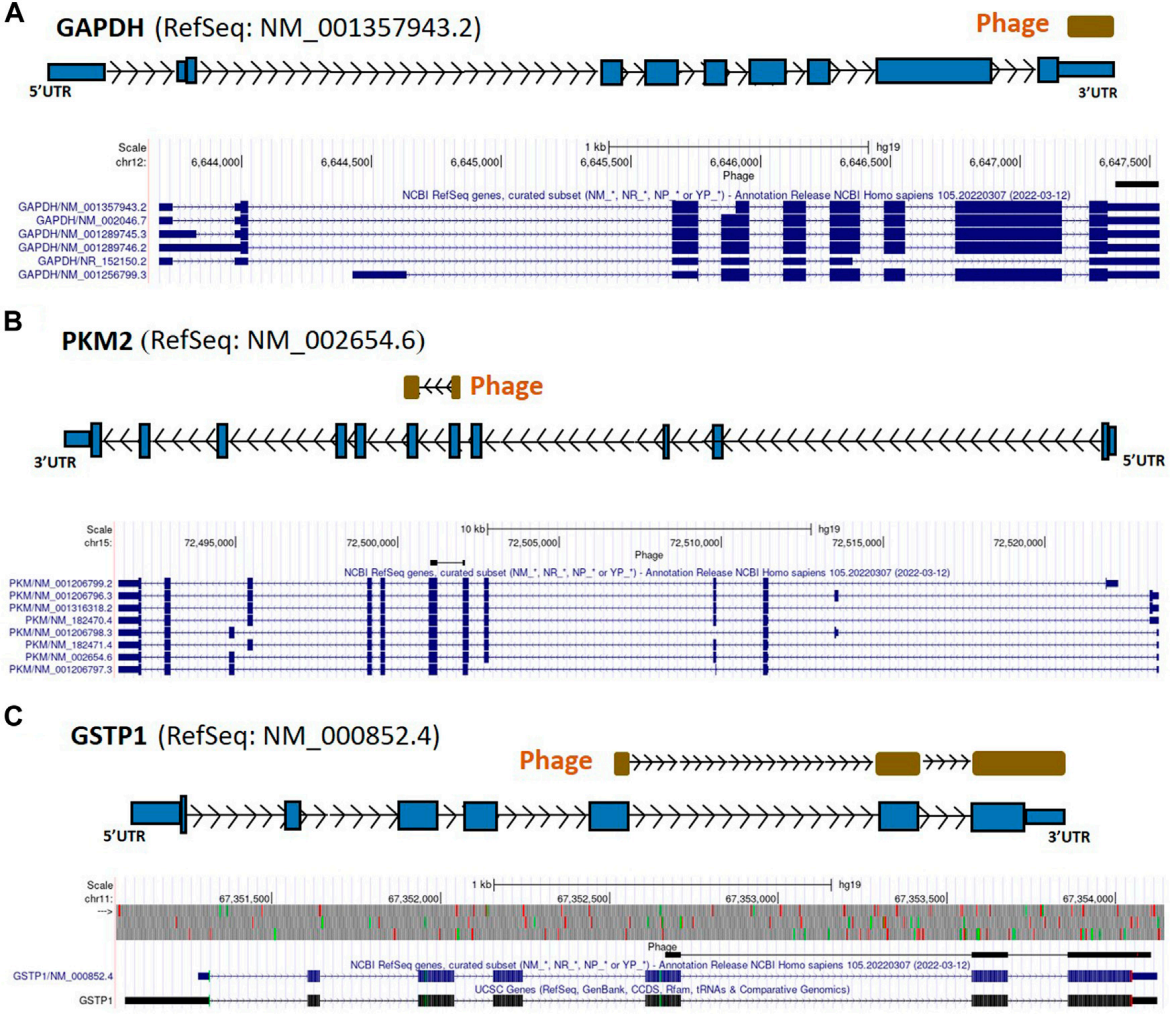
*GAPDH*, *PKM2,* and *GSTP1* gene regions as autoreactive BC antigens identified by UCSC Genome Browser BLAT mapping and annotation of uniquely patient-sera-reactive phages. **(A)** Identified phage mapped completely within the 3′-UTR region of *GAPDH* gene. **(B)** Identified phage mapped to the protein-coding region of *PKM2*. **(C)** Identified phage mapped to the protein-coding region as well as 3′-UTR region of *GSTP1*. *GAPDH*, glyceraldehyde 3-phosphate dehydrogenase; *PKM2*, pyruvate kinase isoenzyme M2; *GSTP1*, glutathione S-transferase Pi 1; UTR, untranslated region; kb, kilobase, EST, expressed sequence tag.

**FIGURE 3:**
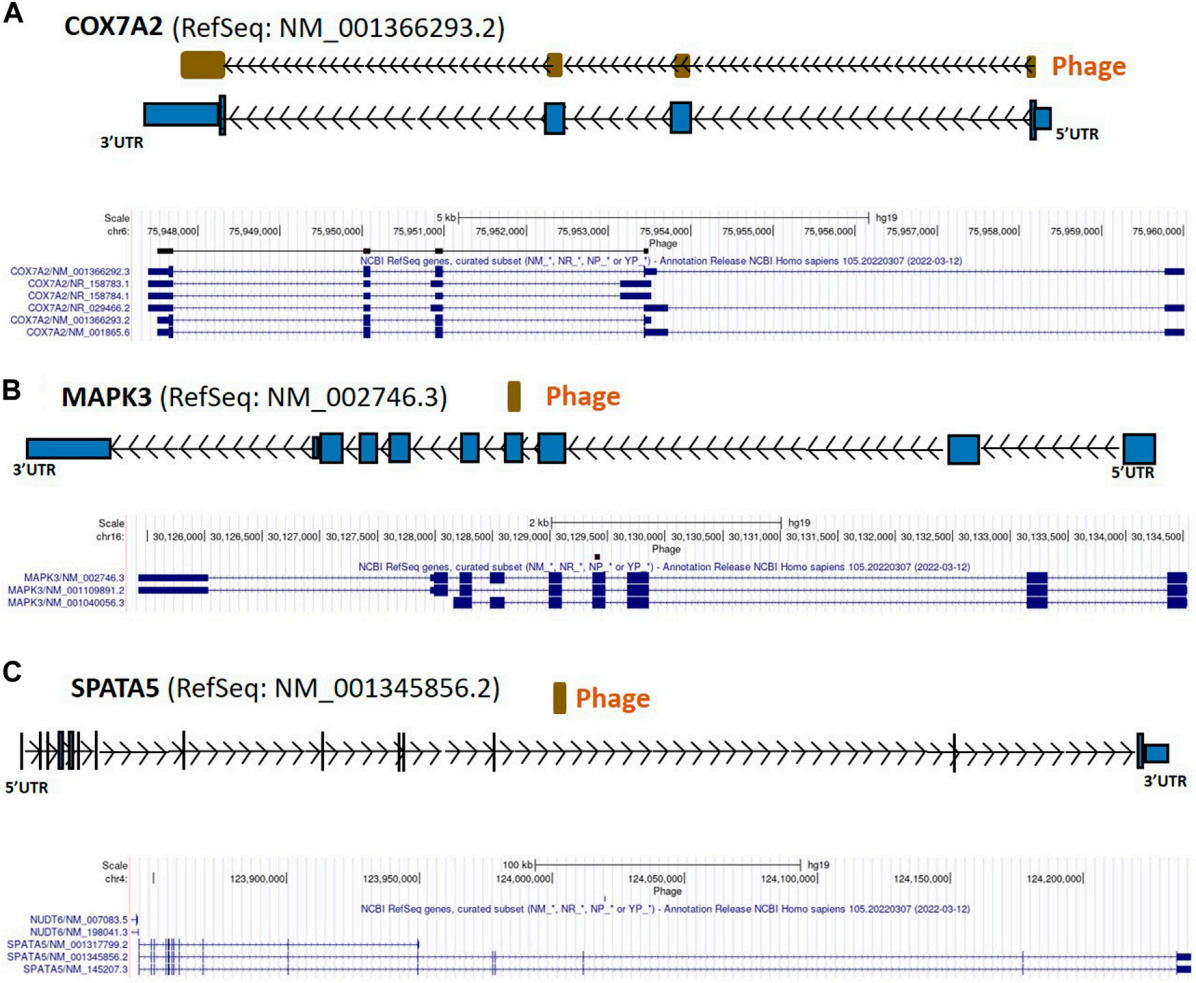
*COX7A2*, *MAPK3*, and *SPATA5* gene regions as autoreactive BC antigens identified by UCSC Genome Browser BLAT mapping and annotation of uniquely patient-sera-reactive phages. **(A)** Identified phage mapping within protein-coding region of *COX7A2* gene. **(B)** Identified phage included protein-coding region of *MAPK3*. **(C)** Identified phage mapped completely within an intron of *SPATA5*. *COX7A2*, cytochrome c oxidase polypeptide 7A2; *MAPK3*, mitogen activated protein kinase 3; *SPATA5*, spermatogenesis associated protein 5; UTR, untranslated region; kb, kilobase.

**FIGURE 4:**
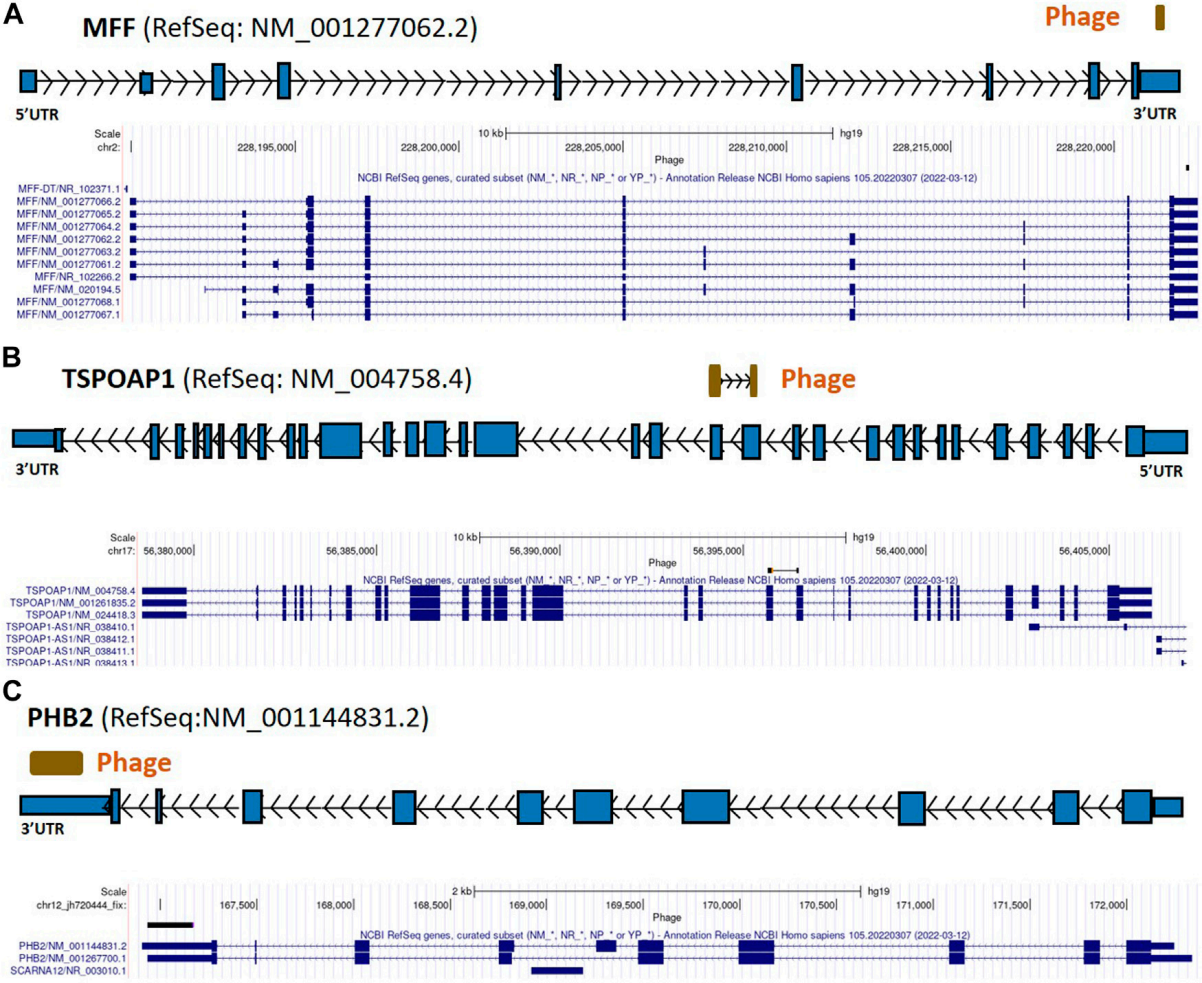
*MFF*, *TSPOAP1*, and *PHB2* gene regions as autoreactive BC antigens identified by UCSC Genome Browser BLAT mapping and annotation of uniquely patient-sera-reactive phages. **(A)** Identified phage mapping within the 3′-UTR region of the *MFF* gene. **(B)** Identified phage mapping to the protein-coding region of *TSPOAP1*. **(C)** Identified phage mapping within the 3′-UTR region of the *PHB2* gene. *MFF*, mitochondrial fission factor; *TSPOAP1*, peripheral-type benzodiazepine receptor-associated protein 1; *PHB2*, prohibitin 2; UTR, untranslated region; kb, kilobase.

**FIGURE 5:**
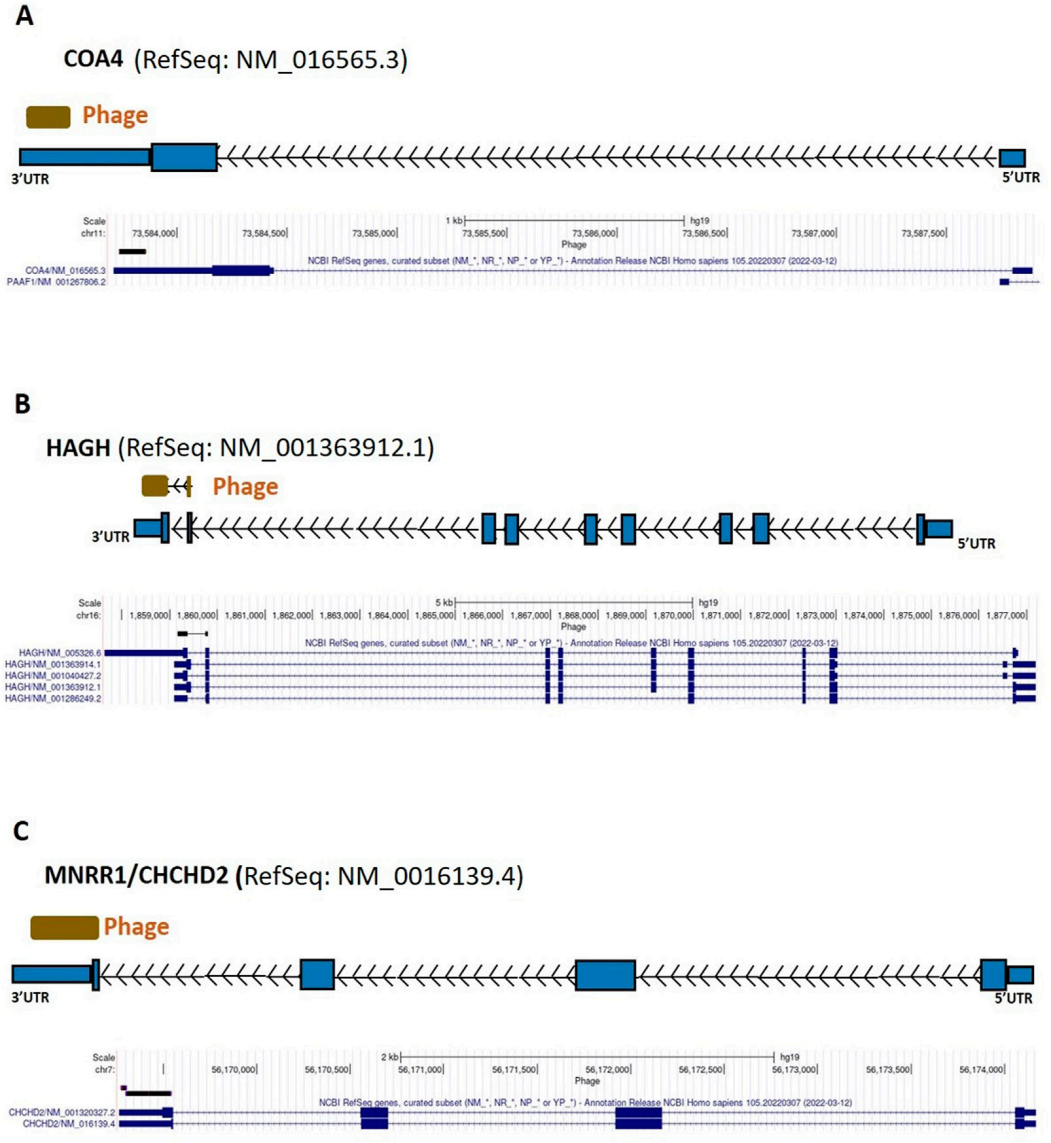
*COA4*, *HAGH*, and *MNRR1* gene regions as autoreactive BC antigens identified by UCSC Genome Browser BLAT mapping and annotation of uniquely patient-sera-reactive phages. **(A)** Identified phage within 3′-UTR region of *COA4*. **(B)** Identified phage included protein coding region as well as 3′-UTR region of *HAGH*. **(C)** Identified phage included protein coding region as well as 3′-UTR region of *MNRR1*; UTR, untranslated region; kb, kilobase; *COA4*, cytochrome C oxidase assembly factor 4 homolog; *HAGH*, hydroxyacylglutathione hydrolase; *MNRR1*, mitochondrial nuclear retrograde regulator 1.

From the 868 phage sequences that immunoreacted with BC sera during immune-screening of cDNA libraries, 547 showing single bands by PCR were successfully mapped to known human genes. Of these, 184 were unique sequences significantly associated with the diagnosis of invasive BC by autoantigen microarray analyses. Although 87 of these sequences were encoded by mitochondrial DNA (mtDNA) (Maroun et al., 2017), 97 were encoded by nDNA, including those mapping to twelve mitochondrial genes (Figure 1): glyceraldehyde 3-phosphate dehydrogenase (*GAPDH*), pyruvate kinase isoenzyme M2 (*PKM2*), glutathione S-transferase Pi 1 (*GSTP1*) (Figure 2), spermatogenesis-associated protein 5 (*SPATA5*), mitogen activated protein kinase 3 (*MAPK3*), cytochrome c oxidase polypeptide 7A2 (*COX7A2*) (Figure 3), mitochondrial fission factor (*MFF*), peripheral-type benzodiazepine receptor-associated protein 1 (*TSPOAP1*), prohibiton 2 (*PHB2*) (Figure 4), cytochrome C oxidase assembly factor 4 homolog (*COA4*), hydroxyacylglutathione hydrolase (*HAGH*), and mitochondrial nuclear retrograde regulator 1 (*MNRR1*) (Figure 5), also called *CHCHD2,* and highlighted in our prior work as an autoreactive mitochondrial gene in BC (Aras et al., 2019) (Table 1; Supplementary Material). From the nDNA-encoded mitochondrial gene products listed in Table 1, ten were cloned from the multi-human BC cell line cDNA library and two from the BC tumor tissue-derived library (Novagen). *GAPDH* and *GSTP1* were represented by three clones each, *MFF* and *PHB2* by two clones, whereas all the rest were single clones. Interestingly, the autoreactive sequences of the nDNA-encoded mitochondrial genes were mapped to their protein-coding regions as well as to non-coding regions, specifically the 3′-UTRs. As indicated in Table 1, autoantibodies in BC sera significantly associated with invasive BC targeted protein-coding exonic regions of the *PKM2* (Figure 2B)*, MAPK3* (Figure 3B)*, COX7A2* (Figure 3A), and *TSPOAP1* (Figure 4B) genes, but only intronic regions of *SPATA5* (Figure 3C). For *GAPDH* (Figure 2A), *MFF* (Figure 4A)*, PHB2* (Figure 4C)*, COA4* (Figure 5A), and *MNRR1* (Figure 5C) genes, only 3′-UTR sequences were recognized as non-self, whereas for *GSTP1* (Figure 2C) and *HAGH* (Figure 5B), the 3′-UTR as well as protein-coding exonic regions all displayed autoreactivity.

**TABLE 1:** NDNA-encoded mitochondrial autoantigens associated with the diagnosis of invasive breast cancer.

Antigen	(p) BC	Accession	cDNA Library	Target
*GAPDH* ^a^	0.001	NR_152,150.2	Multi-cell line	3′ UTR
*PKM*2^a^	0.001	NM_001206799.2	Multi-cell line	Protein-coding region
*GSTP1* **^a^**	0.02	NM_000852.4	Multi-cell line	Protein-coding region, and 3′UTR
*COX7A*2	8.23E-09	NP_00153221.1	Multi-cell line	Protein-coding region
*MAPK*3	0.01	NM_001040056.3	Multi-cell line	Protein-coding region
*SPATA*5^a^	4.94E-06	NG_051570.1	Multi-cell line	Intron
*MFF* ***^a^***	0.001	NR_102,266.2	Tumor tissue-derived (Novagen)	3′ UTR
*TSPOAP*1	0.001	NM_024418.3	Tumor tissue-derived (Novagen)	Protein-coding region
*PHB2*	0.04	NM_001267700.1	Multi-cell line	3′ UTR
*COA4*	0.001	NM_016565.3	Multi-cell line	3′ UTR
*HAGH*	0.05	NM_005326.6	Multi-cell line	Protein-coding region, and 3′ UTR
*MNRR1* ^b^	6E-120	NM_016139.4	Multi-cell line	Protein-coding region and 3′ UTR

^a^ Genes and *p* values were included as unpublished data in a review that appeared prior to this paper; however, the raw data was not published (Fernández Madrid et al., 2020).

^b^ Already published by our group as autoreactive mitochondrial gene in BC (Aras et al., 2019).

*GAPDH*, glyceraldehyde 3-phosphate dehydrogenase; *PKM*2, pyruvate kinase isoenzyme M2; *GSTP1*, glutathione S-transferase Pi 1; *COX7A*2, cytochrome c oxidase subunit 7A2; *MAPK*3, mitogen activated protein kinase 3; *SPATA*5, spermatogenesis associated protein 5; *MFF*, mitochondrial fission factor; *TSPOAP*1, translocator protein, mitochondrial peripheral-type benzodiazepine receptor-associated protein*; PHB,* Prohibiton 2; *COA4*, Cytochrome C Oxidase Assembly Factor 4 Homolog; *HAGH*, Hydroxyacylglutathione Hydrolase. Exons contain protein-coding regions and 3′-UTRs (the last exon of a coding gene contains the end of its protein-coding sequence and all of its 3′-UTR).

**FIGURE 6:**
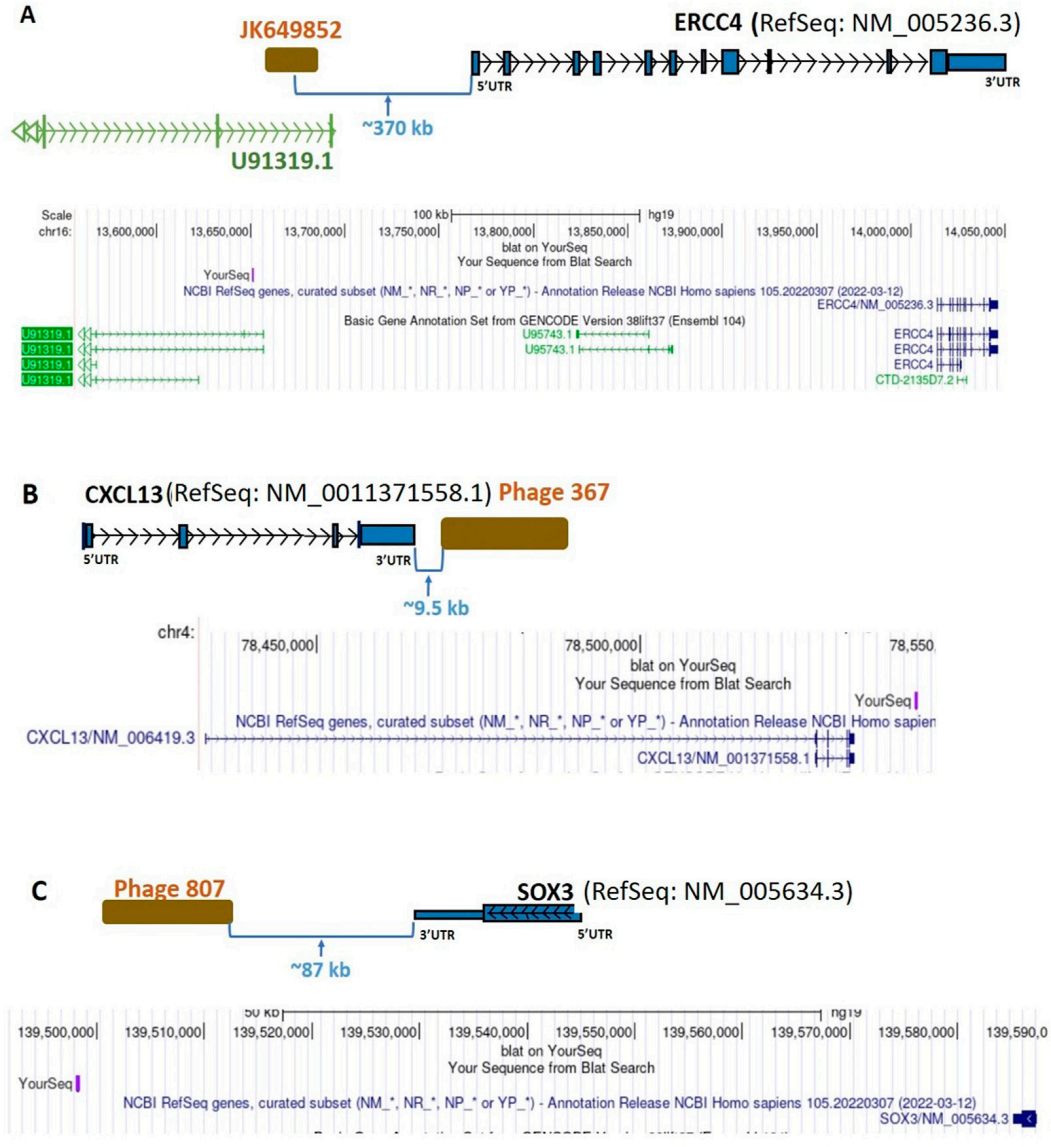
Diagrammatic representation of intergenic sequence mappings of the inserts of uniquely BC-patient-sera-reactive, cDNA-library-derived phages close to the genes *ERCC4*, *CXCL13*, and *SOX3*. **(A)** Phage JK649852, an intergenic phage, close to the coding gene *ERCC4*. **(B)** Phage 367, an intergenic phage, close to *CXCL13* coding gene. **(C)** Phage 807, an intergenic phage, close to the *SOX3* coding gene. *ERCC4*, ERCC Excision Repair 4; *CXCL13*, C-X-C Motif Chemokine Ligand 13; *SOX3*, SRY-Box Transcription Factor 3; UTR, untranslated region; kb, kilobase.

**FIGURE 7:**
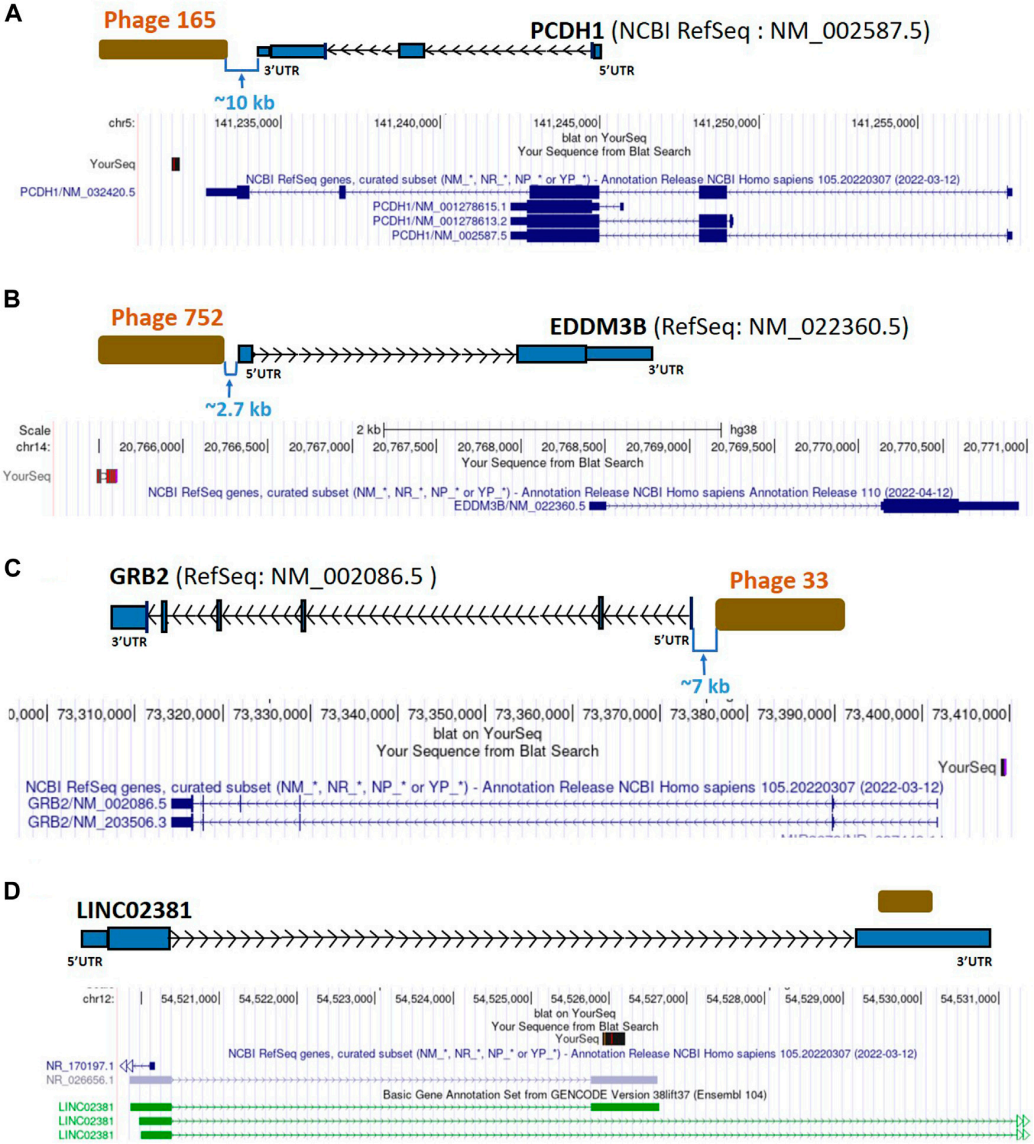
Diagrammatic representation of lncRNA *LINC02381* matching and intergenic sequences from uniquely BC-patient-sera-reactive phages mapping close to *PCDH1*, *EDDM3B*, and *GRB2*. **(A)** Phage 165, an intergenic phage, close to the *PCDH1* coding gene. **(B)** Phage 752, an intergenic phage, close to the *EDDM3B* coding gene. **(C)** Phage 33, an intergenic phage, close to the *GRB2* coding gene and *Y-RNA* gene. **(D)** A hit within the lncRNA *LINC02381*; *PCDH1*, Protocadherin 1; *EDDM3B*, Epididymal Protein 3B; *GRB2*, Growth Factor Receptor Bound Protein 2; UTR, untranslated region; kb, kilobase.

In Figures 2–7, if there is more than one RefSeq gene model per gene due to alternative initiation/splicing/termination and/or database redundancy, the selection of the model to use was based on visual assessment, in the UCSC Genome Browser, of the gene’s underlying mRNAs and ESTs (not shown in these figures) in terms of the numbers of mRNAs and ESTs supporting each transcript model; where mRNA distribution was approximately equal between transcript isoforms, ESTs were used as tiebreakers. Direction of phages (arrow inside phage box) was determined based on the orientation of canonical splice site (GT-AG) and/or polyadenylation (AATAAA, ATTAAA) sequences; if no such sequences were found direction was unknown and phage box is shown without directional arrows.

### Long non-coding RNA and intergenic sequences represented by our library phages are targeted by autoantibodies in breast cancer

Of the 97 nDNA-encoded phage sequences significantly associated with the diagnosis of invasive BC by autoantigen microarray analyses, several clones mapped to the Long Intergenic Non-Protein Coding RNA 2381 (*LINC02381*), and to intergenic phages that may be parts of uncharacterized lncRNAs adjacent to the ERCC Excision Repair 4 (*ERCC4*), C-X-C Motif Chemokine Ligand 13 (*CXCL13*), SRY-Box Transcription Factor 3 (*SOX3*), Protocadherin 1 (*PCDH1*), Epididymal Protein 3B (*EDDM3B*), and Growth Factor Receptor Bound Protein 2 (*GRB2*) genes (Figures 6, 7; Table 2; Supplementary Table S2). The clone representing the putative lncRNA adjacent to *ERCC4* represents the validation of an identical sequence identified using a different, independent collection of BC sera in 2011. That sequence was previously reported by us to GenBank (Phage 1008 = accession number JK649852) (Supplementary Table S2). At that time, little was known about lncRNAs (Kapranov et al., 2007; Spizzo et al., 2012), and the significance of this clone as a potential match of a new non-protein-coding transcript had been unclear to us. *LINC02381* and the intergenic-mapping phage sequences identified as neighbors of *ERCC4, CXCL13, SOX3,*, *EDDM3B* and *PCDH1* were all identified by biopanning the multi-cell line cDNA library, whereas the intergenic-mapping phage sequences identified as neighbors of *GRB2* were cloned from the Novagen library.

**TABLE 2:** LncRNA antigens associated with the diagnosis of invasive breast cancer.

Antigen	(p) BC	Transcript model accession number	cDNA Library
*LINC02381*	0.0007	NR_026656.1	Multi-cell line
lncRNA neighbor of *CXCL13*	2.82e-26	None	Multi-cell line
lncRNA neighbor of *ERCC4*	0.0009	JK649852	Multi-cell line
lncRNA neighbor of *SOX3*	1.04e-08	None	Multi-cell line
lncRNA neighbor of *PCDH1*	4.89E-26	None	Multi-cell line
lncRNA neighbor of *EDDM3B*	5.65E-05	None	Multi-cell line
lncRNA neighbor of *GRB2*	7.00E-09	None	Tumor tissue-derived (Novagen)

*LINC02381*, long intergenic non-protein coding RNA, 2381; *CXCL13*, C-X-C motif chemokine ligand 13; *ERCC4*, ERCC, Excision Repair 4; *SOX3*, SRY-Box Transcription Factor 3, *PCDH1,* Protocadherin 1; *EDDM3B,* Epididymal Protein 3B*; GRB2*, Growth Factor Receptor Bound Protein 2.

## Discussion

### Mitochondrial autoreactivity plays a role in breast carcinogenesis

Multiple nDNA-encoded mitochondrial genes (Table 1) were identified during our immune-screening analysis of BC cDNA libraries. These genes have been linked to energy metabolism, cell proliferation, apoptosis, disease susceptibility, and ribosomal biogenesis (Kim et al., 2017; Abate et al., 2020). Autoreactivity to mitochondrial antigens is an indication of autoimmunity in BC (Fernández Madrid et al., 2004; Aras et al., 2019). The autoreactivity of nDNA-encoded mitochondrial gene products, reported in the present study, provides further evidence for mitochondrial autoimmunity in BC. Specifically, we identified autoreactive *GAPDH* and *PKM2* clones (multi-cell line cDNA library) (Table 1; Supplementary Table S1); these genes play a key role in aerobic glycolysis and apoptosis, while *GAPDH* expression is also associated with BC cell proliferation and tumor aggressiveness (Guo et al., 2013). Further, we previously reported that antibody reactivity to centrosome assembly and/or microtubule proteins was highly associated with the diagnosis of BC (Maroun et al., 2017), suggesting that the autoreactivity might be the expression of autoimmunity developing in breast carcinogenesis. Autoreactivity of the 3′-UTR of *GAPDH* (Figure 2A) as well as of the coding and noncoding regions of *PKM2* (Figure 2B), a gene whose product catalyzes the last step of glycolysis and plays an important role in tumor cell proliferation, may indicate that their gene dysfunction promotes carcinogenesis. *PKM2* is also upregulated in BC tissue, and—consistent with our phage-based screening results—high levels of *PKM2* have been associated with poor prognosis of BC patients (Zhu et al., 2017).

We also identified *MAPK3* as a BC autoantigen (Table 1; Supplementary Table S1). The MAP Kinase pathway is central in cancers, and is interesting in that the *PKM2* and tristetraprolin (TTP) interaction is crucial for the control of breast cancer cell proliferation (Huang et al., 2016). The p38/MAPK pathway might be involved in this regulatory mechanism since treatments with the p38 inhibitor SB203580 or p38 siRNA abolished TTP protein degradation induced by *PKM2* (Huang et al., 2016). Additionally, *PKM2* promotes stemness of breast cancer cells through the Wnt/β-catenin pathway (Zhao et al., 2016). Collectively, several members of the MAPK pathway and fundamental energy metabolism genes are autoantigenic in our screen, and are known from prior work to be directly pertinent to breast cancer carcinogenesis.

In addition, here we found that the protein-coding region and the 3′-UTR of the *GSTP1* gene were targeted by BC autoantibodies (Figure 2C; Table 1; Supplementary Table S1). The *GSTP1* is frequently inactivated in BC and other cancers (Lin and Nelson, 2003), whereas its polymorphisms have been associated with elevated BC risk (Sergentanis and Economopoulos, 2010). Likewise, it partially inhibits the c-Jun N-terminal kinase (JNK)-mediated cell signaling pathway that is part of the MAPK family (Wang et al., 2001) (Morrison, 2012). Downstream of the JNK pathway are the P53 and nuclear factor kappa B (NF-κB) pathways (Buschmann et al., 2000). JNK activation is a cellular response to environmental inflammatory stresses, hence the *GSTP1* being targeted by autoantibodies in BC is compatible with the hypothesis that autoimmune tissue damage in BC leads to chronic inflammation and tumorigenic signals (Fernández Madrid et al., 2015; Maroun et al., 2017; Aras et al., 2019) (Adler et al., 1999). Autoantibodies targeting *GSTP1* and the AP-1 complex suggest that the immune system detects the participation of these signal transduction molecules in breast carcinogenesis.

As additional evidence for the contribution of mitochondrial autoimmunity to breast carcinogenesis, autoantibodies in BC sera were found to target coding and noncoding regions of the *COX7A2* gene (Figure 3A) and 3′-UTR of *COA4* (Figure 5A; Table 1; Supplementary Table S1). COX7A2 is a component of cytochrome *c* oxidase (COX), the last enzyme of the mitochondrial electron transport chain that drives oxidative phosphorylation and has a critical role in regulating energy production in mitochondria, a vital process for cell survival (Zhang et al., 2016). COX7AR (COX subunit VIIa polypeptide 2-like protein), a novel COX subunit recently found to be involved in mitochondrial supercomplex assembly and respiration, is expressed in high energy-demanding tissues (such as aggressive human BC), responds to estrogen (a nuclear hormone that is the key effector of estrogen-responsive ER-positive breast cancer, one of the three fundamental types of breast cancer), and resembles both *COX7A2* and *COX7A1* (Zhang et al., 2016). We found no direct evidence of involvement of *COA4* in BC in the literature but given that it is involved in stabilizing the membrane association of cytochrome *c* (Gladyck et al., 2021), we think that the presence of antibodies against its product may alter the function of COX. The autoreactivity of the mtDNA-encoded enzyme components of complexes I, IV, and V of the catalytic unit (Maroun et al., 2017), and that of *COX7A2* and *COA4*, suggest that the electron transport chain is deregulated in BC.

Our finding of BC-associated autoreactivity of the non-coding region of the *SPATA5* (Figure 3B; Table 1; Supplementary Table S1) suggests that this autoantibody may be a biomarker of BC risk as well as of *SPATA5* dysregulation. *SPATA5* is implicated in ribosome biogenesis (Tafforeau et al., 2013; Prattes et al., 2019), a crucial process for cell growth and proliferation that is often upregulated in cancer cells and plays a key role in the development and progression of most spontaneous cancers (Pelletier et al., 2018).

We also found that autoantibodies in BC sera targeted both coding and non-coding regions of the *TSPOAP1* gene (Figure 4B; Table 1; Supplementary Table S1). This gene encodes a highly conserved protein of the mammalian outer mitochondrial membrane, the peripheral-type benzodiazepine receptor-associated protein 1 (PBR), which was found to be present in aggressive breast cancer cell lines and in human glioma cells, where it may be involved in cell proliferation (Corsi et al., 2008). Its functions include mitochondrial regulation, steroid biosynthesis and import into mitochondria, mitochondrial oxidative phosphorylation, as well as contributions to cell differentiation, proliferation, and apoptosis (Corsi et al., 2008; Batarseh and Papadopoulos, 2010). Thus, the autoreactivity of *TSPOAP1* in BC might reflect its role in apoptosis and cell proliferation.

BC autoantibodies also recognized as non-self the 3′-UTR of *MFF* (Figure 4A). Among pathways related to *MFF*, apoptosis and autophagy are both critical in breast carcinogenesis, (Gandre-Babbe and van der Bliek, 2008; Simula et al., 2017), and there is a connection between BC and mitochondrial fission abnormalities (Tang et al., 2018; Humphries et al., 2020). Here we found that the autoreactivity of *MFF* is localized to the 3′-UTR and does not involve the protein-coding region (Figure 4A; Table 1; Supplementary Table S1). This suggests that the quality control mechanism responsible for mitochondrial fission may be impaired in breast carcinogenesis through dORF-dependent or direct RNA-dependent functions of the *MFF* mRNA.

The 3′-UTR of *PHB2* (Figure 4C) was also targeted by autoantibodies in BC, consistent with the gene’s cancer-relevant roles in oxidative phosphorylation, mitochondrial biogenesis and apoptosis (Signorile et al., 2019). *PHB2* was also found to play a role as an estrogen receptor alpha repressor, which results in an overall negative effect on estrogen-dependent cancers like BC. However, PHB2 can interact with brefeldin A-inhibited guanine nucleotide-exchange protein 3 (BIG3) in BC, which captures PHB2 in the cytoplasm of cancer cells and thereby inhibits the suppressive ability of PHB2 (Yoshimaru et al., 2013). In a similar way, we hypothesize that targeting *PHB2* by autoantibodies disrupts its role as tumor suppressor.

Furthermore, the autoantibodies reacted to protein-coding and 3′-UTR regions of *HAGH* (Figure 5B), part of a pathway to detoxify methylglyoxal (MGO). The role of MGO in cancer is controversial because lower doses of MGO are able to establish an adaptive response in cancer cells while higher doses cause cellular apoptosis (Leone et al., 2021). We think the alteration of *HAGH* as a result of autoantibody targeting plays a role in influencing the role of MGO in BC. Finally, consistent with our data on MNRR1 autoreactivity in breast carcinogenesis (Aras et al., 2019), we found that autoantibodies in BC sera targeted the 3′-UTR region of *MNRR1* (Figure 5C).

### Protein-coding as well as non-coding regions of nuclear genomic DNA-encoded mitochondrial genes are targeted by autoantibodies in breast cancer

Little is known about the immunogenicity of nucleic acids in cancer and about the possibility that direct autoreactivity of RNA moieties, in addition to that of peptides encoded by the RNAs’ ORFs, may deregulate the function of important gene products involved in carcinogenesis. Previous reports outlined that RNA molecules can form surfaces that mimic those of proteins and can trigger reactivity of autoantibodies with RNA surfaces due to cross-reactivity between a protein epitope and the RNA (Keene, 1996). Conformational RNA epitopes were discovered because autoantibodies in the sera of patients with systemic autoimmune diseases react directly with discrete structural elements in the Ul RNA of the small nuclear ribonucleoproteins (snRNPs), transfer RNA (tRNA) and ribosomal RNA (rRNA) (Deutscher and Keene, 1988; Uchiumi et al., 1991). More recent reports showed numerous autoreactive RNA sequences in the RADs in humans and in animal models of systemic lupus erythematosus (SLE), as well as in viral infections (Tan, 1982; Suurmond and Diamond, 2015). Although many types of RNA sequences are normally untranslated, they play an important role in regulating gene expression in multiple ways. For instance, introns contain numerous transcriptional regulatory elements, may encompass enhancers or alternative promoters, can regulate alternative splicing, affect the transcript stability or half-life, and/or modulate the efficiency of mRNA translation (Shaul, 2017). The 3′-UTR is involved in numerous regulatory processes including transcript cleavage, translation, and mRNA subcellular localization (Navarro et al., 2021). In addition, in our previous work we have proven that some of these 3′-UTR regions sequences are translated in human cells and in BC (Bánfai et al., 2012; Choo et al., 2022).

Intronic autoreactivity, such as what we observed in *SPATA5* (Figure 3C), in theory may be a consequence of intron retention from aberrant or incomplete splicing, followed by translation of the intron-encoded continuation of the gene’s ORF or a secondary intronic ORF. This process may yield a protein even though that protein is truncated and/or out-of-frame in reference to the mRNA’s translation product. Deregulation of alternative splicing is thought to be important in aggressive BC (Yang et al., 2019). Splicing could also play a protective role against genetic instability in eukaryotic cells by preventing R-loop accumulation (Bonnet et al., 2017); this suggests a possible mechanism by which the autoantibodies targeting introns can influence carcinogenesis. As opposed to the role of uORFs in gene regulation, the role of dORFs is still unclear; recent reports suggested they may enhance translation of the canonical ORF and, depending on the number of dORFs in the gene, this effect varies (Wu et al., 2020). Our identification of immunoreactive sequences from the 3′-UTR regions of several genes (Figures 2, 6; Table 1) indicates that the autoantibodies target either the mRNA directly or the translated peptides of the dORFs, suggesting that dORF translation from these genes’ mRNAs may be immunogenic in BC. Mutations in the 3′-UTR may mediate oncogene activation or tumor suppressor inactivation by altering microRNA (miRNA/miR)-binding efficiency to the miRs’ specific cognate sites (Nicoloso et al., 2010). Frequent somatic mutations in the 3′-UTR of *GAPDH* have been reported to promote the growth of ovarian cancer by sponging miR-125 and thereby affecting the expression of Signal Transducer and Activator of Transcription 3 (STAT3) (Liu et al., 2020). Here we found that autoreactivity of the 3′-UTR of *GAPDH* (Figure 2A) is highly associated with the diagnosis of invasive BC (Table 1) and we speculate that, similarly to the findings in ovarian cancer (Liu et al., 2020), changes in the 3′-UTR responsible for autoreactivity may disturb the binding of a miRNA to the 3′-UTR of the *GAPDH* mRNA. We also found that the 3′-UTR of *MFF* (Figure 4A) is targeted by autoantibodies in BC sera. It is possible that the expression of *MFF* in BC may be influenced by the binding of miRNAs to its 3′-UTR. MiR-376b-3p can attenuate mitochondrial fission and cardiac hypertrophy by targeting the 3′-UTR of *MFF* (Sun et al., 2018). Moreover, members of the miR-376 family have been implicated in breast and ovarian cancers, gliomas, and leukemia, and in the regulation of autophagy. This is potentially important because dysregulation of autophagy is a key feature of BC and other cancers (Zhang et al., 2018).

Our results point to direct immune autoreactivity of protein-coding gene 3′-UTRs and introns *in vitro*, without participation of immune cells or the direct influence of the microenvironment. This suggests that the autoimmunity drivers in BC may affect the epithelial cells in the breast early in carcinogenesis. Since immune-screening of the multi-cell line library used RNA from established BC cell lines for cDNA library construction, the recognition of the cloned gene products as non-self also suggests the possibility that cancer epithelial cells, in addition to being able to produce immunoglobulins, can highjack to some extent the process of antigen presentation. The autoreactivity of the mtDNA-encoded enzyme components of the ETC catalytic unit and that of *COX7A2* along with *COA4* suggests that, even though mitochondria often behave normally in assays, the transfer of electrons may be altered in BC. In addition, the large number of autoreactive mitochondrial gene products generated in cancer epithelial cells without participation of immune cells suggests inherent abnormalities of mitochondria in BC.

### Non coding RNA dysregulation as a consequence of autoreactivity may play a role in breast carcinogenesis

Many tumor antigens are not detected by the immune system because they are not adequately presented by dendritic cells (Apavaloaei et al., 2020). The lncRNA, and the 3′-UTR and intronic regions that we report here were cloned from the multi-breast cancer cell line cDNA library made with RNA from BC cells devoid of signals from the tumor environment. We know that these antigens are recognized by the immune system since they trigger an autoantibody response. However, although epithelial cancer cells are poor antigen presenting cells of major histocompatibility complex (MHC)-associated peptides, our data indicate that cancer cells are involved in some way in the presentation of these lncRNAs to the immune system as indicated by the reacting autoantibodies. We don’t know how cancer epithelial cells present these immunogenic RNA sequences to immunocompetent cells, and this question deserves further investigation.

Anti-nucleic acids autoantibodies are hallmarks of autoimmune rheumatic diseases: anti-DNA antibodies are characteristic of systemic lupus erythematosus (SLE) while anti-RNA autoantibodies commonly occur in SLE and Sjögren syndrome, as well as in viral diseases (Blanco et al., 1991; Yoshimi et al., 2012). Despite multiple reports of autoantibodies in cancer sera, to our knowledge autoantibodies targeting lncRNA have not been previously reported. Our finding is significant and novel because, although dysregulation of lncRNA associated with various diseases including breast cancer has been extensively reported (Suvanto et al., 2020), we found no reports of dysregulation of lncRNAs induced by immune autoreactivity. We also have identified multiple intergenic phages that are part of ncRNAs and we are unclear whether they would be part of previously unrecognized lncRNAs.

In this work, we identified *LINC02381* located ∼50 kb away from Single-strand selective monofunctional uracil-DNA glycosylase (*SMUG1*) as a BC antigen highly associated with the diagnosis of BC (Table 2; Supplementary Table S2). *LINC02381* has attracted considerable attention for its strong links with the development of solid tumors including BC and autoimmune diseases. It is downregulated in gastric cancer (Jafarzadeh and Soltani, 2020) and is part of a signature of immune-related lncRNAs in BC (Ghafouri-Fard et al., 2021). *In vitro* studies indicated that *LINC02381* may function as a tumor suppressor by regulating the phosphatidylinositol 3-kinase (PI3K-AKT) signaling pathway (Jafarzadeh et al., 2020) and more recently this was confirmed *in vivo* where a knockdown of the gene was found to hinder proliferation, migration and invasion of BC cells, while overexpression promoted carcinogenesis (Huang et al., 2022). PIK3CA mutations, as well as AKT activation by phosphorylation (pAKT), are detected in many cancers, especially in BC (Yang et al., 2016). Thus, autoreactivity of *LINC02381* may be clinically significant by impairing its ability to regulate PI3K-AKT. As mentioned above, as a breast cancer autoantigen *LINC02381* inhibits gastric cancer progression by regulating Wnt signaling (Jafarzadeh and Soltani, 2020), a highly active pathway in cancer cells (Kazi et al., 2016). A further link between BC and rheumatic autoimmune diseases was suggested by the report that *LINC02381* can exacerbate rheumatoid arthritis through adsorbing miR-590-5p and activating the mitogen activated protein kinase signaling pathway (Wang and Zhao, 2020). For example, *LINC02381* has been shown to accelerate cancer progression in osteosarcoma, cervical cancer, and glioma (Chen et al., 2020; Bian et al., 2021; Sun et al., 2021), and to function as a tumor suppressor in breast and gastric cancers (Jafarzadeh and Soltani, 2020; Ghafouri-Fard et al., 2021). Since *LINC02381* exacerbates rheumatoid arthritis (Wang and Zhao, 2020), its discovery as a BC autoantigen provides another example of a lncRNA exerting effects at the interface of cancer and autoimmune diseases. The low expression of SMUG1 was also associated with poor survival in ER positive BC and improved survival in ER- BC patients, suggesting that it plays a variable role in BC and can be used to predict response to therapy (Abdel-Fatah et al., 2013).

In the present study, four phage clones mapped to an intergenic sequence, possibly representing a previously uncharacterized lncRNA neighbor of the *CXCL13* gene (located ∼9.5 kb away from the 3’ end of *CXCL13*) (Figure 6B), were targeted by autoantibodies in BC sera (Table 2; Supplementary Table S2). The inflammatory chemokine CXCL13 is associated with the chronic inflammatory conditions severe rheumatoid arthritis (Bugatti et al., 2014) and multiple sclerosis (Krumbholz et al., 2006). The autoreactivity-induced deregulation of *CXCL13* in BC reported here supports existing work reporting on chronic inflammation and cancer development (Coussens and Werb, 2002; Mantovani et al., 2008). Additionally, it is known that *CXCL13* is overexpressed in BC (Panse et al., 2008) and associated with BC progression, poor prognosis and metastases (Biswas et al., 2014). One possible mechanism is that CXCL13-C-X-C Motif Chemokine Receptor 5 (CXCR5) co-expression may regulate epithelial to mesenchymal transition of BC cells during lymph node metastasis (Biswas et al., 2014). However, this chemokine has paradoxically been associated with improved outcome in patients with the luminal-human epidermal growth factor receptor 2 subtype (Razis et al., 2020), and in patients with HER-2 associated BC (Razis et al., 2012). Nonetheless, our analysis of the ENCODE Transcription Factor ChIP-Seq dataset in the UCSC Genome Browser revealed the *CXCL13*-neighbor phage sequence contains an experimentally-validated consensus binding site for the transcription factor FOXA1, a key mediator of hormonal response in breast cancer (Robinson and Carroll, 2012). The finding that an intergenic non-coding sequence near the *CXCL13* gene is highly associated with diagnosis of BC suggests that autoreactivity of this sequence regulates the adjacent *CXCL13* gene.

Autoantibodies in BC targeted four clones of a non-coding sequence (GenBank accession: JK649852, a phage from our earlier work) that map to an ultra-conserved non-coding region in UCSC Genome Browser’s phyloP analysis of MultiZ 100-species genome alignments and is located ∼370 kb upstream of the *ERCC4* gene (Figure 6A). The sequence also overlaps the intronic region of the lncRNA gene *U91319* (Table 2; Supplementary Table S2), although it does not belong to that lncRNA’s transcriptional unit. This lncRNA is part of an immune-related lncRNA signature predicting survival of uterine corpus endometrial carcinoma patients (Sun et al., 2021), potentially suggesting a broad role for it in the disorders of the female reproductive system. In addition, *U91319* was hypermethylated in astrocytomas, the most common type of glioma (Bendahou et al., 2020). Interestingly, we have identified glioma mutation factor *β* (GMF-β) as an autoantigen associated with the diagnosis of BC (*p* = 0.02). GMF-β promotes glial differentiation. Neurogenesis and vasculogenesis share common regulators with gliomagenesis, suggesting that GMF-β is important in glioma progression *via* promoting neovascularization (Kuang et al., 2015). The phage sequence targeted by BC autoantibodies maps near the *ERCC4* gene (Figure 6A), which is involved in Nucleotide Excision Repair (NER) pathway and has been associated with risk of BC in a 2-stage case-control study in Spanish and Finnish populations and that variation of the gene was associated with a protective effect (Milne et al., 2006). It is very interesting that NER is particularly targeted by autoantibodies in BC. We have reported that the Ku antigen, which plays a critical role in mammalian DNA double-strand breaks repair, is recognized by some patients with the scleroderma-polymyositis overlap syndrome (Mimori et al., 1990) and has been widely implicated in tumor biology, is a breast cancer autoantigen (Fernández Madrid et al., 2004). Other studies have implicated Ku as a key DNA damage repair protein in breast cancer (Alshareeda et al., 2013). We have also reported that the RPA32 subunit of replication protein A (RPA) is targeted by autoantibodies in BC (Tomkiel et al., 2002). The DNA-binding protein RPA plays a major role in DNA metabolism and is an essential component of NER. The data that we report here on the possible link between ERCC4 and BC together with our previous findings of autoreactive Ku antigen and the central role of RPA in DNA replication, recombination, and repair suggest that autoimmunity to *ERCC4*, Ku, and RPA32 reflects molecular changes involved in the process of tumorigenesis, in particular on the importance of NER in breast carcinogenesis.

We also identified an intergenic phage located approximately 87 kb from the nearest coding gene, *SOX3* (Figure 6C; Table 2; Supplementary Table S2). Several studies reported the association of the SOX regulator family with BC (Mehta et al., 2019), and the progression of solid tumors and metastasis (Grimm et al., 2020). SOX3p is expressed in regulatory T cells (Tregs) and has a role in the control of autoimmunity and maintenance of transplantation tolerance (Rudensky, 2011). Our previous studies (Fernández Madrid et al., 2004; Maroun et al., 2017; Aras et al., 2019) indicate that specific mtDNA-encoded and nDNA-encoded mitochondrial gene products, nDNA-encoded signal transduction molecules, and lncRNAs are BC autoantigens. The finding of autoreactivity of intergenic phage neighbor to *SOX3* further supports the contention that autoimmunity-driven deregulation of multiple protein-coding and lncRNA genes is mechanistically involved in breast carcinogenesis. SOX3 acts as a master regulator for regulatory Treg development and function and is indispensable for the maintenance of immunological self-tolerance and homeostasis (Rudensky, 2011; Morikawa and Sakaguchi, 2014).

An intergenic sequence located ∼10 kb away from the 3’ end of the *PCDH1* gene (Figure 7A; Table 2; Supplementary Table S2) was also identified as non-self. *PCDH1* has been studied in asthma patients and was upregulated during the development of airway epithelial barrier. This suggests that *PCDH1* may has a role in the physical barrier against environmental exposures (Kozu et al., 2015). *PCDH1* expression decreased in medulloblastoma, giving patients poor prognosis (Neben et al., 2004); we found no reports of an association with BC. As of now, there is no clearly identified mechanism of how this gene or its product could be associated with cancers. Disruption of this gene by autoreactivity, possibly through the lncRNA neighbor to *PCDH1*, may alter the response to environmental antigens that modulate tumor-immune interactions in BC.

Located ∼2.7 kb upstream of *EDDM3B,* another intergenic-mapping phage sequence was identified as a BC autoantigen target (Figure 7B; Table 2; Supplementary Table S2). *EDDM3B* plays a role in the synthesis and secretion of epidydimal epithelial cells proteins and male fertility (Damyanova et al., 2013). This gene is predictive of late metastasis in node negative BC (Schmidt et al., 2012). However, data about *EDDM3B* in BC are still lacking.

Another intergenic-mapping phage sequence ∼7 kb upstream of *GRB2* was recognized as non-self (Figure 7C; Table 2; Supplementary Table S2). This gene is also close to other known non-coding RNA genes, including a *Y-RNA* gene (19 kb), *RNU6-938P* (3.6 kb) and *miR-3678* (7 kb) (Supplementary Material). The abnormal activation of GRB2 by HER2 (human epidermal growth factor receptor 2) signaling is strictly related to the development of BC (Daly et al., 1994; Ijaz et al., 2017). Circulating Y-RNA fragments with known cellular functions may participate in breast carcinogenesis and have potential as circulating biomarkers (Gulìa et al., 2020) (Dhahbi et al., 2014).

Finally, the dysregulation of multiple lncRNAs (Prensner and Chinnaiyan, 2011; Hu et al., 2018), as well as genetic alterations in single nucleotide polymorphisms (SNPs) (Yan et al., 2015; Chandra Gupta and Nandan Tripathi, 2017; Miao et al., 2018) in cancer, collectively contribute to the global deregulation of the cancer genome, which has been described as “genome chaos” (Heng and Heng, 2020). Future studies should address the validation of our coding, lncRNA, and intergenic hits in additional patient cohorts as well as the elucidation of the potentially drug-targetable molecular mechanisms by which these autoreactive sequences may contribute to breast cancer pathogenesis.

## Conclusion

Several nuclear DNA-encoded mitochondrial gene products recognized as autoantigens*—MNRR1*, *GAPDH, PKM2, GSTP1, COX7A2, MAPK3, SPATA5, TSPOAP1, MFF, PHB2, COA4,* and *HAGH—*in addition to their protein-coding regions, often feature their non-coding regions and 3′-UTRs detected in our phage-based screening. These genes are likely to be involved in breast carcinogenesis through direct immune autoreactivity and may be valuable biomarkers of aggressive BC. *GAPDH, PKM2, GSTP1,* and *SPATA5,* as well as the lncRNA *LINC02381* and the intergenic phages neighbors of *ERCC4, CXCL13, SOX3,* and *EDDM3B* (all cloned by biopanning the multi-cell line library), were autoreactive in epithelial cancer cells *in vitro* suggesting the early participation of autoimmunity in breast carcinogenesis. Autoimmunity to ERCC4 may underscore the pivotal involvement of nucleotide excision repair in breast carcinogenesis. The *SPATA5* gene’s intronic autoreactivity may indicate BC risk, and its known participation in ribosome biogenesis may be an etiologic link to breast carcinogenesis. This work shows that, although autoreactivity of key mitochondrial gene products is involved in breast carcinogenesis, other non-mitochondrial pathways can also be oncogenic. It is important to note that these are results of an *in vitro* study and specific to our population in Detroit, and further *in vivo* studies would be necessary to confirm these findings. Many lncRNAs are deregulated in carcinogenesis (Suvanto et al., 2020) and have the potential to influence multiple oncogenic pathways but the reasons behind the deregulation of lncRNAs in cancer are poorly defined. Here we add autoreactivity as a potential cause that may explain part of the aberrant regulation of lncRNAs that is observed in carcinogenesis.

## Data Availability

The datasets presented in this study can be found in the GenBank database (https://www.ncbi.nlm.nih.gov/genbank/). The accession numbers can be found in the Supplementary Material. Further inquiries can be directed to the corresponding authors.

## Glossary

**BC**: Breast Cancer
**nDNA**: nuclear genomic DNA
***GAPDH***: glyceraldehyde 3-phosphate dehydrogenase
***PKM2***: pyruvate kinase isoenzyme M2
***GSTP1***: glutathione S-transferase Pi 1
**SPATA5**: spermatogenesis associated protein 5
**MFF**: mitochondrial fission factor
**TSPOAP1**: translocator protein
**PBR**: peripheral-type benzodiazepine receptor-associated protein
**UTR**: untranslated region
**IFA**: Immunofluorescence
**BBD**: Benign breast disease
**RAD**: rheumatic autoimmune disease
**AMA**: anti-mitochondrial antibody
**PBC**: Primary biliary cholangitis
**PV**: Pemphigus vulgaris
**ETC**: electron transport chain
***MNRR1***: mitochondrial nuclear retrograde regulator 1
**ncRNA**: non-coding RNA
**ORF**: open reading frame
**uORF**: upstream open reading frame
**dORF**: downstream open reading frame
**smORF**: small open reading frame
**cDNA**: complementary DNA
**IDC**: infiltrating ductal carcinoma
**HFHs**: Henry Ford Health System
**TAA**: Tumor Associated Antigen
**PCR**: Polymerase Chain Reaction
**BLAST**: Basic Local Alignment Search Tool
**BLAT**: BLAST-like alignment tool
**GO**: Gene ontology
**mtDNA**: mitochondrial DNA
***COX7A2***: cytochrome c oxidase subunit 7A2
**MAPK3**: mitogen activated protein kinase 3
**miRNA**: MicroRNA
**STAT3**: Signal Transducer And Activator of Transcription 3
**TTP**: Tristetraprolin
**AP-1**: Activator Protein-1
**JNK**: c-Jun N-terminal kinase
**MAPK**: mitogen activated stress kinase family
***PHB2***: Prohibiton 2
***COA4***: Cytochrome C Oxidase Assembly Factor 4 Homolog
***HAGH***: Hydroxyacylglutathione Hydrolase
**MGO**: methylglyoxal
**lncRNA**: long non-coding RNA
**SNP**: single nucleated peptide
***LINC02381***: Long intergenic non-protein coding RNA 2381
***CXCL13***: C-X-C motif chemokine ligand 13
**CCR4-NOT**: Carbon Catabolite Repression—Negative On TATA-less
**PI3K-AKT**: Phosphatidylinositol 3-kinase
**mTOR**: mammalian target of rapamycin
**CDCA4**: Cell Division Cycle Associated 4
**CBX5**: Chromobox 5
**CXCR5**: C-X-C Motif Chemokine Receptor 5
**TFH**: T-Follicular helper cell
***SOX3***: SRY-Box Transcription Factor 3
***PCDH1***: Protocadherin 1
***EDDM3B***: Epididymal Protein 3B
***GRB2***: Growth Factor Receptor Bound Protein 2
**CTLA-4**: Cytotoxic T-Lymphocyte Associated Protein 4
**CTNNBIP 1**: Catenin Beta 1
