# Supplementary material for: Non-coding regions of nuclear-DNA-encoded mitochondrial genes and intergenic sequences are targeted by autoantibodies in breast cancer
Source: Front Genet. 2023 Mar 29;13:970619. doi: 10.3389/fgene.2022.970619 (PMC10111166; doi:10.3389/fgene.2022.970619)
Supplement: Supplementary file 1 [file DataSheet1.docx]

**Supplementary Data**

**1.Further information about culture of BC cells and cDNA T7 library construction**

1.1. Culture of breast cancer cells

Breast cancer cells in culture were required to obtain protein extracts for immunoblots of breast cancer proteins and as a source of mRNA for cDNA library construction. We obtained the seven breast cancer cell lines listed in the manuscript from Dr. Steven Ethier and the pre-malignant cells from Dr. Frederick Miller, both at the Karmanos Cancer Institute.

1.2. Multi breast cancer cell line cDNA T7 library construction

The total RNA was extracted and the mRNA was purified from the MCF-7, T47D, DCIS, SKBR3, SUM44, SUM102, SUM149, and SUM159 cell lines following the manufacturer’s instructions. mRNA purification and cDNA synthesis were completed (Figures 1 and 2).

***
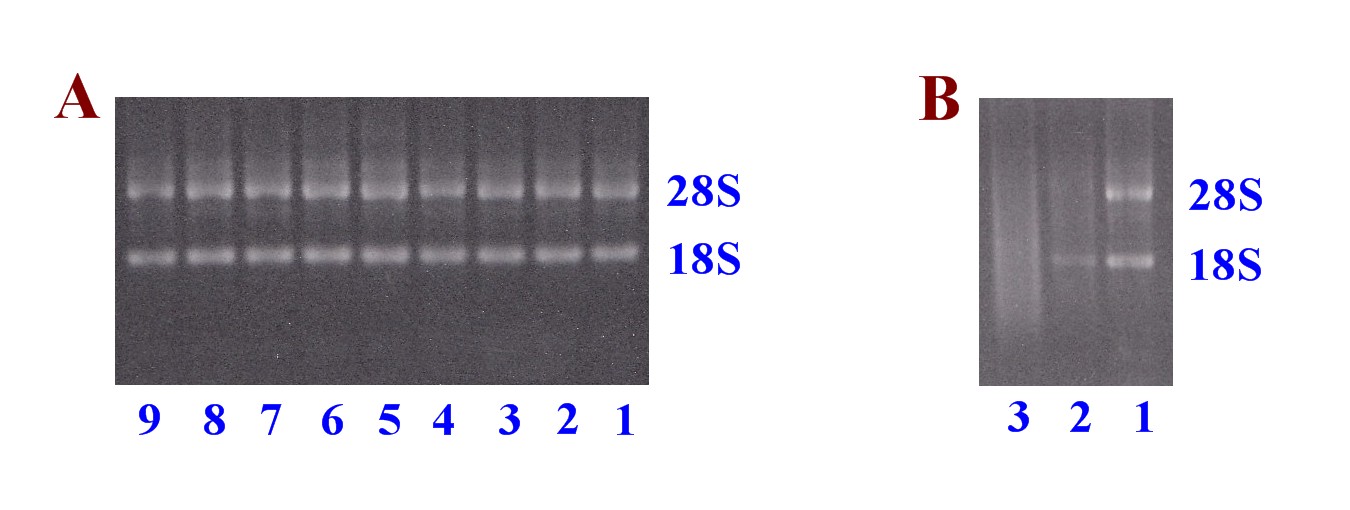
***

Figure 1. Total RNA and mRNA isolated from eight breast cancer cell lines.

A: shows the total RNA including the 28S and the 18S ribosomal RNA from eight breast cancer cell lines: 1, SUM 44; 2, SUM 102; 3, SUM 149; 4, SUM 159; 5, DCIS; 6, MCF-7; 7, SKBR; 8, T47D; 9, total RNA mixture of eight breast cancer cell lines. Loading amount of each lane is 1 µg.

B: shows results from mRNA purification from 2 mg total RNA mixture from eight breast cancer cell lines: 1, total RNA mixture; 2, first round mRNA isolation; 3, second round mRNA isolation. Loading amount of each lane: 1, 1µg; 2, 0.5µg; 3, 1 µg


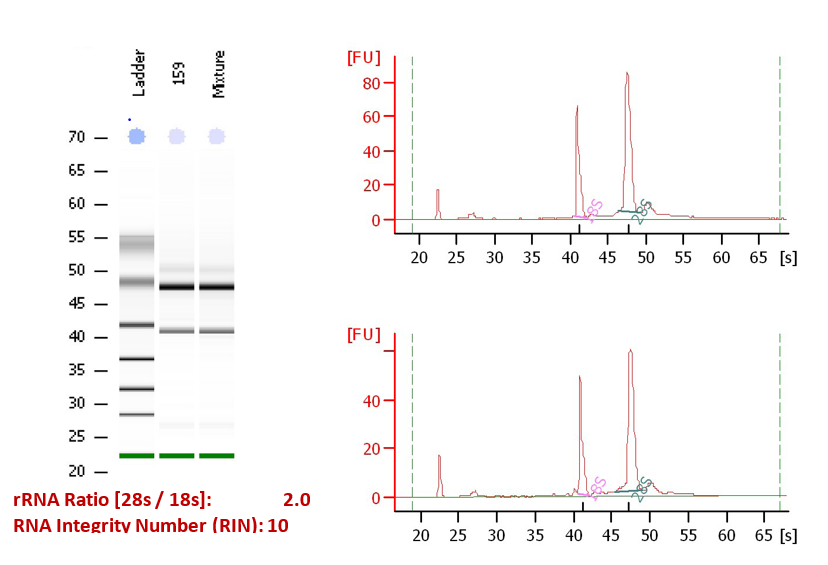


Figure 2. Purity and integrity of total RNA Isolated form eight breast cancer cell lines.

The ratio of 28S to 18S rRNA and RIN values were obtained by analyzing 1μl purified total RNA using an RNA LabChipKit and the Agilent 2100 Bioanalyzer.


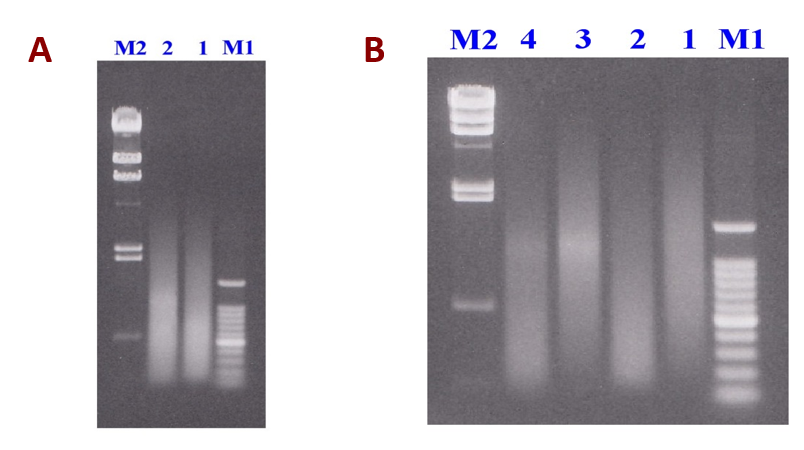


Figure 3. cDNA synthesis from mRNA.

A, indicates cDNA was synthesized using random primers (lane 1) or oligo d(T) (lane 2). Loading amount of each lane is 500 ng. B, shows the EcoR I /Hind III linker ligated-cDNA purified by gel filtration: lane 1. void fractions of random primer-synthesized cDNA; lane 2. additional 250 μl elution fractions after void fractions; lane 3, void fractions of oligo d(T)-synthesized cDNA; lane 4, additional 250 μl elution fractions after void fractions; Loading amount of each lane is 250 ng. Lane M1, 100 bp DNA ladder; Lane M2, lambda DNA/Hind III Marker.

**
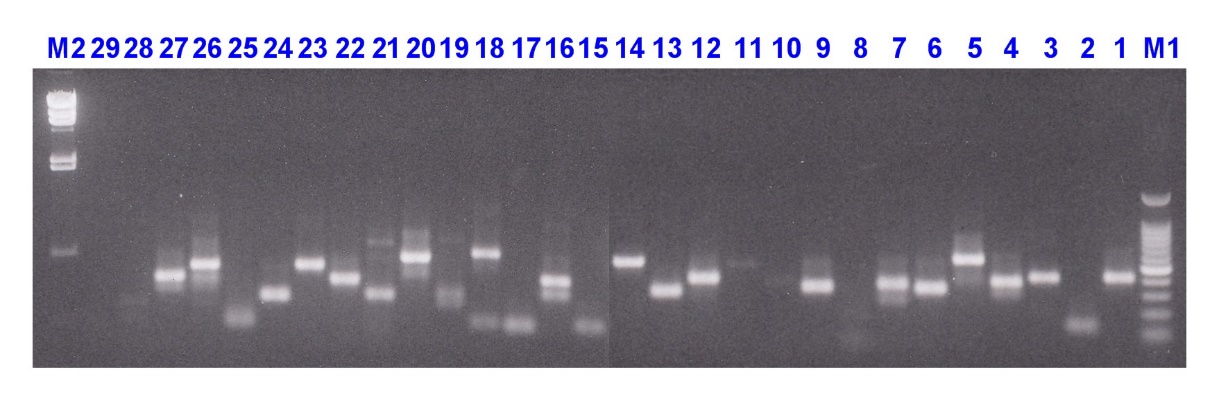
**

Figure 4. PCR amplification of plaques.

Lanes (1-28) PCR products from individual plaques; lane 29, control with water; Lane M1, 100 bp DNA ladder; Lane M2, lambda DNA/Hind III Marker.

Library construction, packaging and identification were performed. Subsequently, the library was used for biopanning with the breast cancer sera selected for immunoscreening.

**2. Supplementary Tables**

**Supplementary Table I.** Identity of nDNA-encoded mitochondrial antigens recognized as autoantigens.

| ***Antigen*** | ***Evalue/ID%*** | ***Library*** | ***Chromosome*** |
| --- | --- | --- | --- |
| *GAPDH* | 5e-81/100% | Multi-cell line | 12p13.31 |
| Sequence: ACAAGAGGAAGAGAGAGACCCTCACTGCTGGGGAGTCCCTGCCACACTCAGTCCCCCACCACACTGAATCTCCCCTCCTCACAGTTGCCATGTAGACCCCTTGAAGAGGGGAGGGGCCTAGGGAGCCGCACCTTGTCATGTACCATCAATAAAGTACCCTGTGCTCAA | | | |
| *PKM*2 | 9E-149/100% | Multi-cell line | 15q23 |
| Sequence: TGGTGGAAGTGGGCAGCAAGATCTACGTGGATGATGGGCTTATTTCTCTCCAGGTGAAGCAGAAAGGTGCCGACTTCCTGGTGACGGAGGTGGAAAATGGTGGCTCCTTGGGCAGCAAGAAGGGTGTGAACCTTCCTGGGGCTGCTGTGGACTTGCCTGCTGTGTCGGAGAAGGACATCCAGGATCTGAAGTTTGGGGTCGAGCAGGATGTTGATATGGTGTTTGCGTCATTCATCCGCAAGGCATCTGATGTCCATGAAGTTAGGAAGGTCCTGGGAGAGAAGGGAAAG | | | |
| *GSTP*1 | 3E-70/99.75% | Multi-cell line | 11q3.2 |
| Sequence: TTGGTCCTGGAGAAAGGAAGGCAAACTCTGCCTCCCGCTTAGAGTCCCCCCAACCCTCACTGTTTCCCGTTGCCATTGATGGGGAGGTTCACGTACTCAGGGGAGGCCAGGAAGGCCTTGAGCTTGGGCCGGGCACTGAGGCGCCCCACATATGCTGAGAGCAGGGGGAACGCATCCAGGCAGCCAGGGGCTAGGACCTCATGGATCAGCAGCAAGTCCAGCAGGTTGTAGTCAGCGAAGGAGATCTGGTCTCCCACAATGAAGGTCTTGCCTCCCTGGTTCTGGGACAGCAGGGTCTCAAAAGGCTTCAGTTGCCCGGGCAGTGCCTTCACATAGTCATCCTTGCCCGCCTCATAGTTGGTGTAGATGAGGGAGATGTATTTGCAGCGGAGGTCCTCC | | | |
| *COX7A*2 | 4E-59/99.77% | Multi-cell line | 6q14.1 |
| Sequence:  ATGGTAAAGACTGCTTTATTGGTGGCAGTTACTACAAGTCAATAAATATTGATCCCCAAAGAAGAGCTCGGTTATTTATCAGATTACTGGTCCATAGAGAGCTGAATGAAACTGAACCAAGCGATTGCTGGGATGACTGAAGTCACTCCTGCTTCTTGGGAAATGAAGCCACAGCCAGCTCATATATGGCATATGCTGTTCCACCAACTGTAAGAATCATAGTGGCTCTATACAGGAGGGCATCAGCTACCCCACCCTTTAGATACAGTGGAATTTCATCATCCTCCTGGAACAGTTTTTGCTTCTCCGGAACTTTATTTTTAAAATGCCTGCGGGAAGCAGTGCTTATCGTCCTCTGCCCAATCTGACGAAGAGCCAGCAGATTCCGCAGCATCTTGGCTGTTACTGACCAGCAACCGCCACAAC | | | |
| *MAPK*3 | 1e-11/96.15% | Multi-cell line | 16p11.2 |
| Sequence:  CTCCGGGGCCGGTACCAGCGCGTAGCCACATACTCCGTCAGGAAGCCGGTG | | | |
| *SPATA5* | 6e-102/100% | Multi-cell line | 4q28.1 |
| Sequence:  TTCAGATATTAGAGAAAATAGTGTGGAAGTTCCTAAAAAATTTAAAATAAAACTATCTTATGATCCAGCAATTCCACTATTGAGTATATATCCAAAGGAAATGAAATCAGTATGTCCAAGAGACATCTGCACTCCATGTTCACTGCAGCATTATTCACAATATCCAAGTCATGGAATCAACCTAAGTATGCATCGAAGAATAAGCG | | | |
| *MFF* | 5E-38/100% | Tumor tissue-derived (Novagen) | 2q36.3 |
| Sequence:  GAAAGTGTTAATGTTGAAGTGTCGATGCAAAAATGTCTATTAACAAAACTGCTTAACACTGTTTGAGAAACAGGACAAAATAATTGTTTTTC | | | |
| *TSPOAP1* | 8e-102/99.06% | Tumor tissue-derived (Novagen) | 17q22 |
| Sequence:  GTGGATGGACTCGGAGTGGGAGGAGTTGGAGAGAGACTCTGCCTTCTTGGCTGTCCTTCGAGGGACCCCAGTGAGAGTGGCAGGGGCAGGCTCGGAAGACTTTGGGGTGCAGCCGCCCAGGGGAGCCCNGGCGGGAGGTCAAGGTCTTTGGGGCCAGACCCCCGGCAAGGCTGGGGAATGGAGCAGCAGCAGGGGGGTGGTGGGCAGTCTCCA | | | |
| *PHB2* | 2e-120/100% | Multi-cell line | 12p13 |
| Sequence:  TCAGGTTAAGTAATAAAAATTTATTGAGAATTCCTGGGTTGGTGTTTATCTCCTCCCAGCCTTGAGGGAGGGAACAACACTGTAGGAAATCACTGAGAAATCACGCACTGTCCCCAACAGCCCCAGTTAACACAGGGAGGAGGAAAGTAATTCCCCAGAAAAGGGGCTAGTCTTCAGTCTTCCTTAATCCAAGAGGGGTTCAGGGAACCGGTGTGGGGGACCATCGCATGATACTGGGCTTGAATTCGGATCCCCGAGCATCACACCTGACTGGAATACGACAGCTCCCNAAAATTGTTACTNCAGTGTTCCCGTCAGATTTTTTAGCAAAAAACCAG | | | |
| *COA4* | 3e-52/99.2% | Multi-cell line | 11q13.4 |
| Sequence:  TNNTTTTCCTTCCACTAAACTGATTGAAACATCCAGAACAAGTGATGAAAGAGAAAGGCACTAATTAGGTAATCAATTTACCATTAGATTGTAAATTTAATTTAAAATAAAATGTCACTAAAACA | | | |
| *HAGH* | 3e-51/100% | Multi-cell line | 16p13.3 |
| Sequence:  CCACAGTGCCATCCACCCTGGCAGAGGAGTTTACCTACAACCCCTTCATGAGAGTGAGGGAGAAGACGGTGCAGCAGCACGCAGGTGAGACGGACCCGGTGACCACCATGCGGGCCGTGCGCAGGGAGAAGGACCAGTTCAAGATGCCCCGGGACTGAGGCCGCCCTGCACCTTCAGCGGATTTGGGGATTAGGCTCTTTTAGGTAACTGGCTTTCCTGCTGGTCCGTGCGGGAAATTCAGTCTTGATTTAACCTTAATTTTACAGCCC | | | |
| \| *MNRR1* \| 6e-120 \| Multi-cell line \| 7p11.2 \| \| --- \| --- \| --- \| --- \| | | | |
| *Sequence:*  TGCCAAGGGGTTAACTAAGTTACTCGAGTGCGGCCGCAAGCTTTGTGAGTTAATTAGAATAAAATCATAACATAACAGCTAGTTTAAGGAGGCCACACAAACATTTGCCCAGTCCCAGATTCTACAGAGTAGGGACACCCCCACTTCCATTTCAATTCTGAAGCAAGGAAGCTAGGAATGACAGGAGAGGTTTAACTGATGGTTACACTTTATACCCTCACTATCAATTCTATTTTTATACTAAATTAACTTAGTTATGAGAGCTGATTTTCCATCTCTCCAGGTTGAACTTCTTGCTTGAATTCGGATCCCCGAGCATCACACCTGACTGGAATACGACAGCTCCAAGGCTTGAATTCGGATCCCCGAGCATCACACCTGACTGGAAAGTCTGGATCCACATGGCTGGACATAATTTAACTAAGCCAGAAACTGGCTACTCTAAAAGCTAAATAAGGAAGCCCGCTTTCATGTCC | | | |

***Legend.*** *GAPDH, glyceraldehyde 3-phosphate dehydrogenase; PKM2, pyruvate kinase isoenzyme; M2;GSTP-1, glutathione S-transferase Pi 1; COX7A2,cytochrome c oxidase subunit 7A2; MAPK3, mitogen activated protein kinase 3; SPATA5, spermatogenesis associated protein 5; MFF, mitochondrial fission factor; TSPOAP1, translocator protein, mitochondrial peripheral-type benzodiazepine receptor-associated protein; PHB2:Prohibiton 2; COA4 Cytochrome C Oxidase Assembly Factor 4 Homolog; HAGH:* *Hydroxyacylglutathione Hydrolase; MNRR1: Mitochondrial Nuclear Retrograde Regulator 1*Multi-cell line, cDNA library constructed with RNA extracted from established BC cell lines; Tumor tissue-derived, cDNA library of commercial origin (Novagen).

**Supplementary Table II.** LncRNA antigen sequences associated with the diagnosis of invasive breast cancer.

| ***Antigen*** | ID% | Accession | Chromosome |
| --- | --- | --- | --- |
| *LINC02381* | 98.97% | NR_026656.1 | 12q13.13 |
| Sequence: GACTTGCTTCGCAGGGATCTGGGAAGCTCAGCCGGCAGAGCTGAGAGCCGCAGTTGCATCCTGGAGCCTGATGCTAGAAGCAGCTTCCGTCTTTGGGTTCTTGCTGCNTCGGCCTCTGCTCTGTTCAGTTTGCTGTTGTGTTTTTCTCCCCCATGTTGGGGTGGTGGGGTACAGGGAAATAAAATGCTTTCTCCCAGGCCCCTAATCCTTCCCCATGCCTCCATCAGCCTCAAAGCTGCTGACAGTCATGAACTGCACCTTCCAGCCCTGCCCATAAGCTACTCAAAGCAAA | | | |
| *lncRNA neighbor of CXCL13* | 96.77% | none | 4q21.1 |
| Sequence: GGACGATTCAGCTGCCAGTCCTAGAACACAAAAAAGCAAAGAGGAAAAGCAAATAGAAACCTGCCAGTACTGGAACACTGAGAAGTAAACAGAAAAAGCAAACTAAAAAAATTGCTTCCAAGATTTTGCTCACAACCTGAGATGATACAAGAAAAGGACTTAAAAAAAAAAAAAAAAAAAAAAAAAACCCAAACTTGGGGGCCCCCCCCAATAAACAANTAACCCCCTGGGGCCCCCAAACGGGGCCTGGGGGGTTAAATAAAAATCCCGGGGGGNGGCNCNTAGCCCGGGCNGGNATGTTTTTGGTTTTTTTTTTTNTGTCTTTACGNG | | | |
| *lncRNA neighbor of ERCC4* | 96.9% | JK649852 | 16p13.12 |
| Sequence: CGGCCGCAAGCTTTGCCACAGGTGTCTTCTAATCATCATTTATTCCAGTACTCAAATGTCCTGTTATTCACCAGGCTGAGCTTCTCCTTAGTGGCGTTAAAGGGACATTGGGTTTGGTTGAAATTCCCGGCAAATACCTTTTTCACACAAATGCATTGCATGGAAATTTGAACATTGTATTTTTGGTCTAATTATCTGAATCANGATCCCCGAGCATCACACCTGACTGGAATA | | | |
| *lncRNA neighbor of SOX3* | 100% | none | Xq27.1 |
| Sequence:  CGGCCGCAGCTTTGAGTANTTAGACAGGCAGGCAGGCCAAGGTAGGCCTGGGCCAGCTGAGGAGCAGATGGCTGCAGGAGCAGCAGCTGGGGCCCATCCATAGGCATGTCTCTAGCCTGCCTCTGAGCTCTGGATAAAACTTCTAAGGGTCACAGAACAAGAAGCTCTGTGCTGTGTGATTGATTGATTGATTTAGAAATGGACAGATTTTTTTTTAAAGCAACTAATGC | | | |
| *lncRNA neighbor of PCDH1* | 99.55% | none | 5q31.3 |
| Sequence:  TTAGGCTGGTAGCCCTGGCATACGAAGCCCTTCTACAGAGGGGAAGGGGTAGAATGGGATGGGGTGGCAGCAGCCAGGTCTAGGGGTTGGCCAAAGGCTCCTTTCCCTTGGGGAGGCCCAGGAGATGGGCTTGGGGCTTGTTAAAGAGACAGCCCCTTTCCCCTGCACAAATGGGGCAGGCGCAATAGTCTTGGAGTTGGTAGTATTGGGTGCCCAGCTGGTGGAACAGTGGCCTCAAGG | | | |
| *lncRNA neighbor of EDDM3B* | 81.7% | none | 14q11.2 |
| Sequence:  TTTATTTATTTATTTATTTATTTTTTTTTTTTTTTTNNNGGNGNANTCCCCCTCGGNCCCCCGGGGGGGAGGGCAGGGGCGCAATCTCGGCTCACTGCAAGCTCTGCCCCCCAGGTTCAACCCATTCC | | | |
| *lncRNA neighbor of GRB2* | 99.8% | none | 17q25.1 |
| Sequence: CAAGCTTCTCTTTAGGGGGAAAGCACTAGATACCCAGTAACAGCCAGTGACAGCCTAAAATTGGGCATTTGAAATTCTATGCTCCCTTGCCCAATTGATCTTGGAAGTTCTCATCTAGAATTTCCTTCACTTAAGTTGTGATCCAAGGCTGTGTACTCCTCTCAGGCTTCCAGTGTTCCCTCTGATGCTGAGTTTAGCAATGAGACCAAGTGCTGTGACCCCTCTCCAACATCCTGGCCACCTTTTCCTTCCTTCCTTCCTTCTTTCCTTCCTTCCTGCCTTCCCTCCCTCCCTCTTTCTTTCCTTCCTTCCTTCTTTCTTTTTTCTTTCTTTCCTTTCTTTTTCCTTTCATTTTCTTTTTTTCTCTTTCTTTCTTCATTTTCTTTCTTTCTCTCTCTTTCTCTCTTCCTTTCTTTACCTCCTTTCTTTCCTCTCTTTCTCTTTATCTCTCCCTCTTTCTCTCG | | | |

**Legend**. LINC02381, Long Intergenic Non-Protein Coding RNA 2381; CXCL13, C-X-C Motif Chemokine Ligand 13; *ERCC4*, *ERCC Excision Repair 4*; SOX3, SRY-Box Transcription Factor 3; PCDH1, Protocadherin 1; EDDM3B, Epididymal Protein 3B; GRB2, Growth Factor Receptor Bound Protein 2.

**3.Genome browser results**

**UCSC Genome Browser results for *GAPDH.***


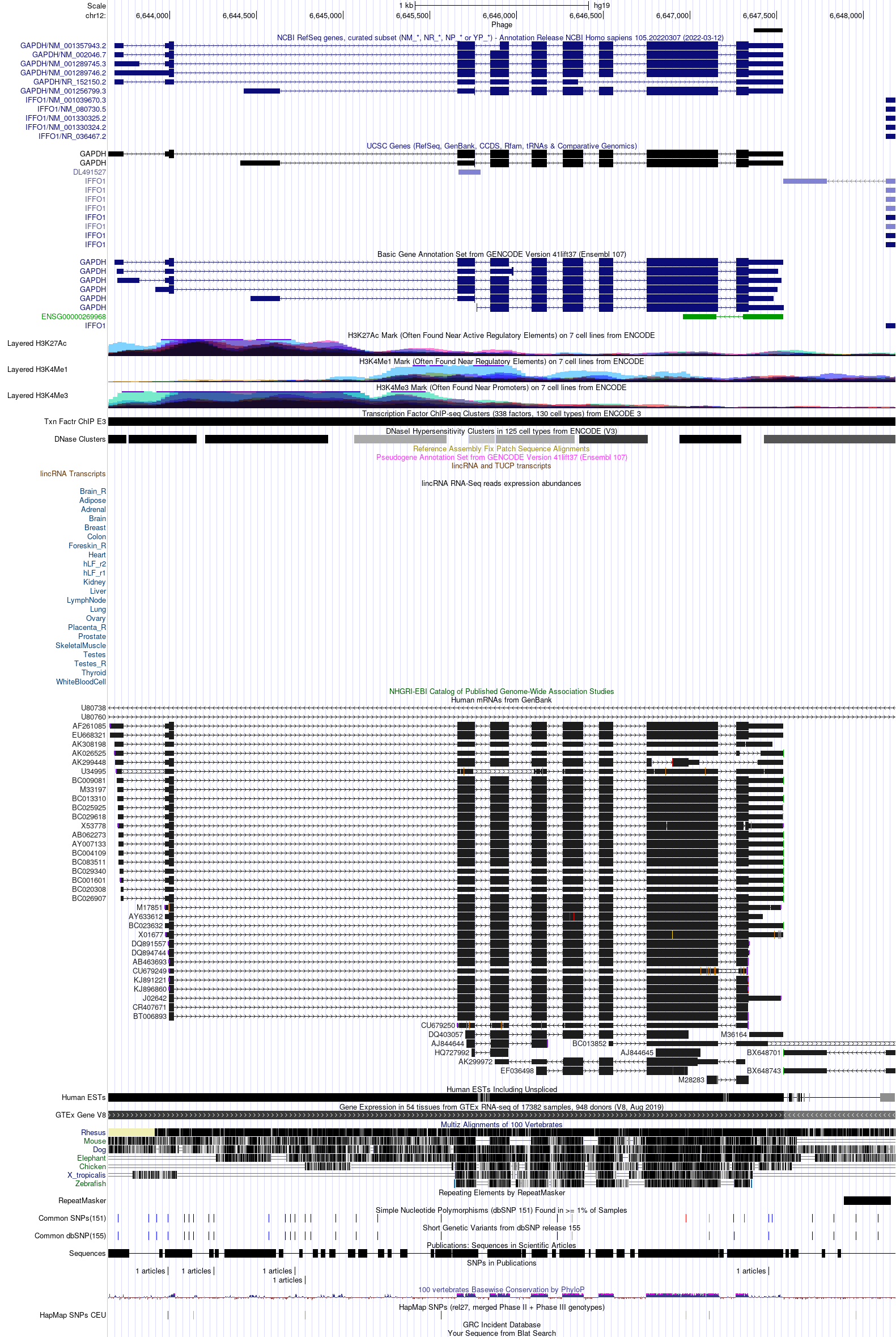


**UCSC Genome Browser results for *PKM2.***


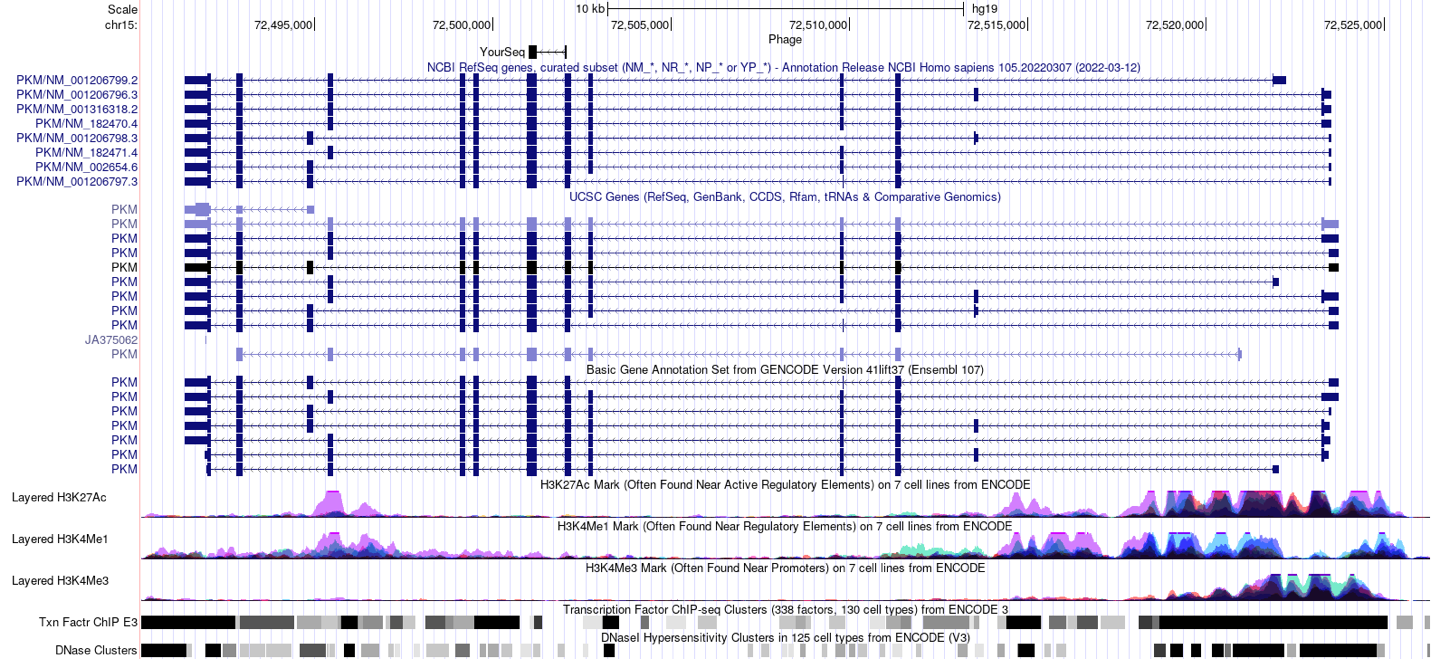


**UCSC Genome Browser results for *GSTP1.***


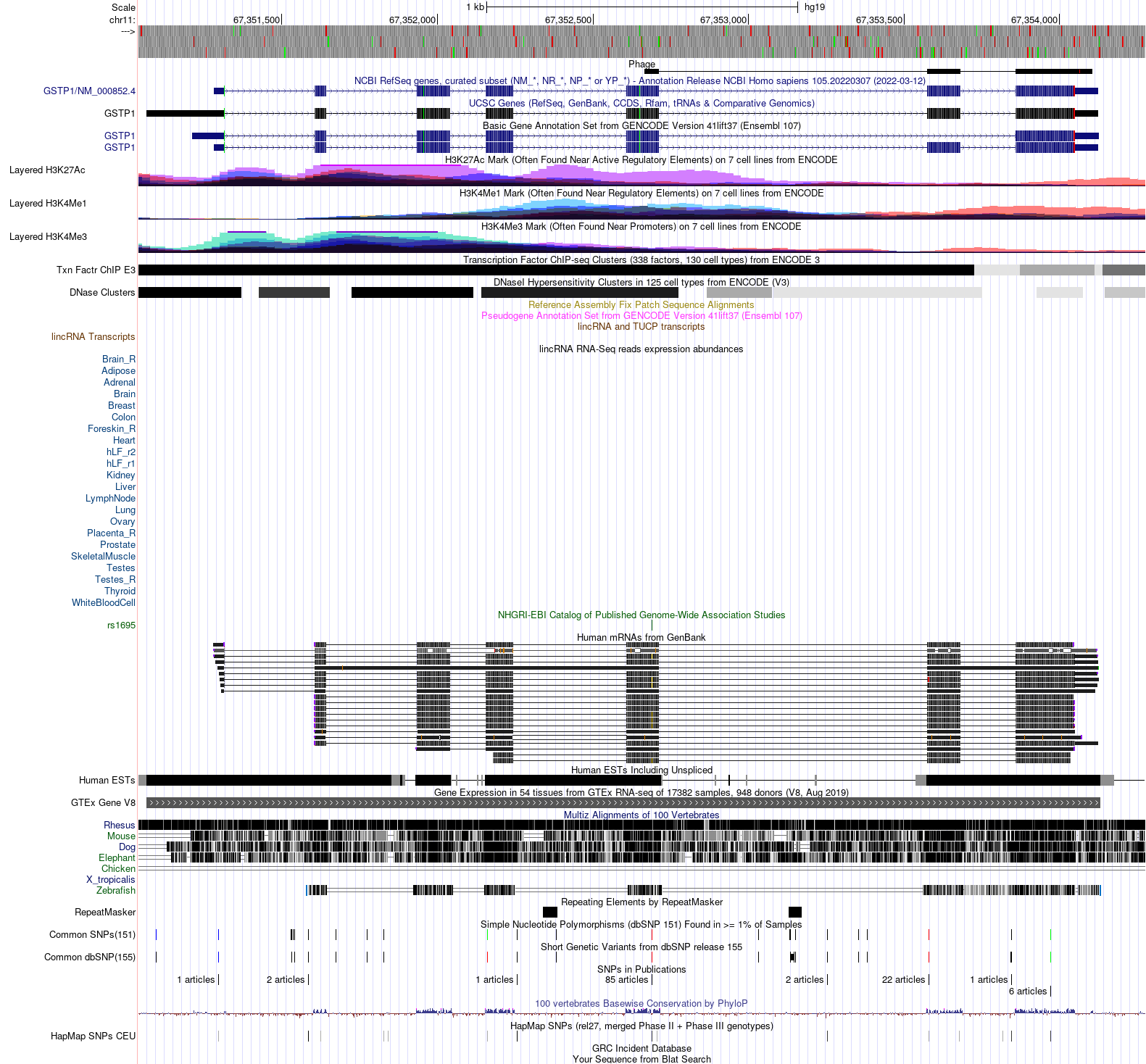


**UCSC Genome Browser results for *COX7A2.***


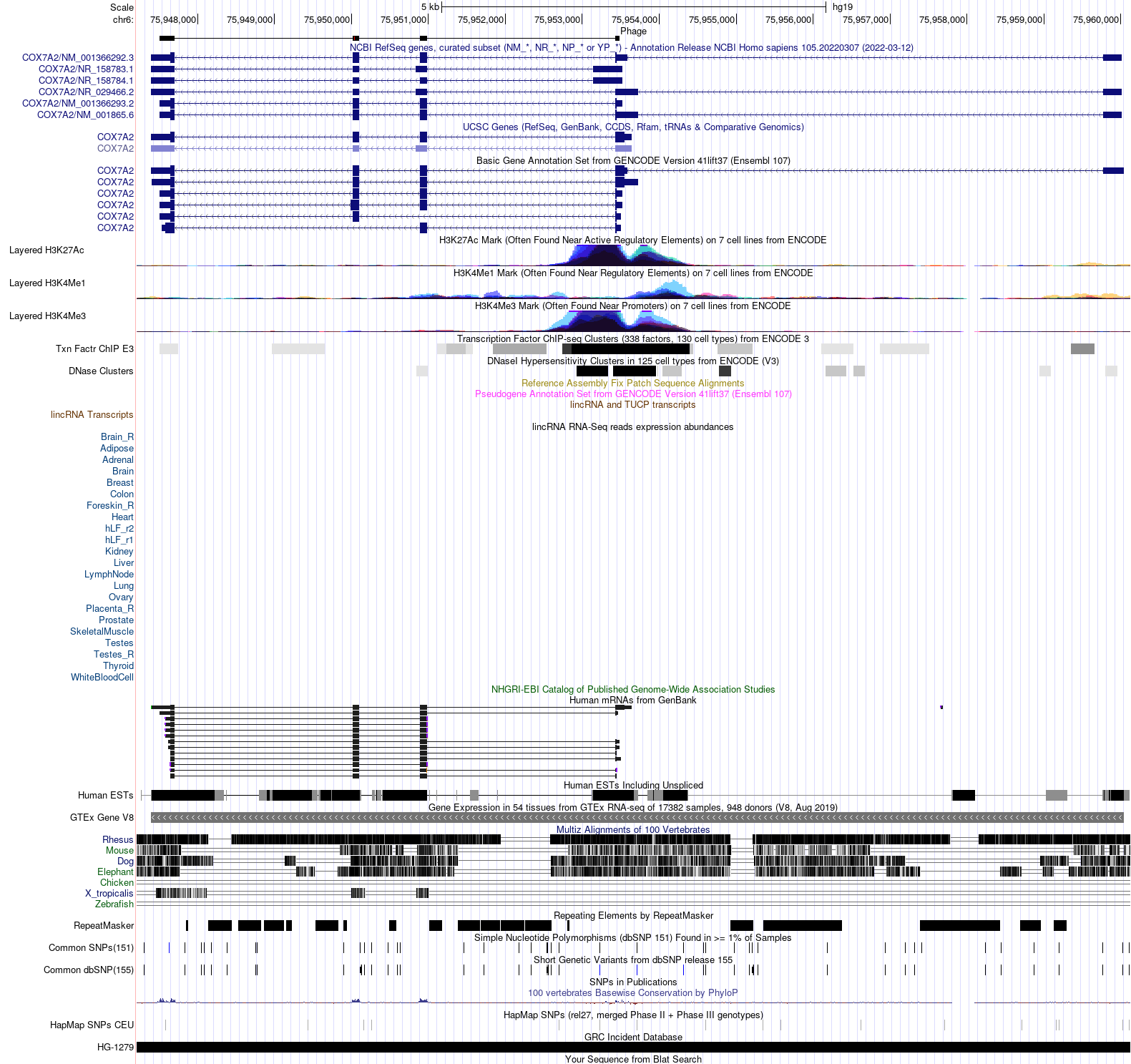


**UCSC Genome Browser results for *MAPK3.***


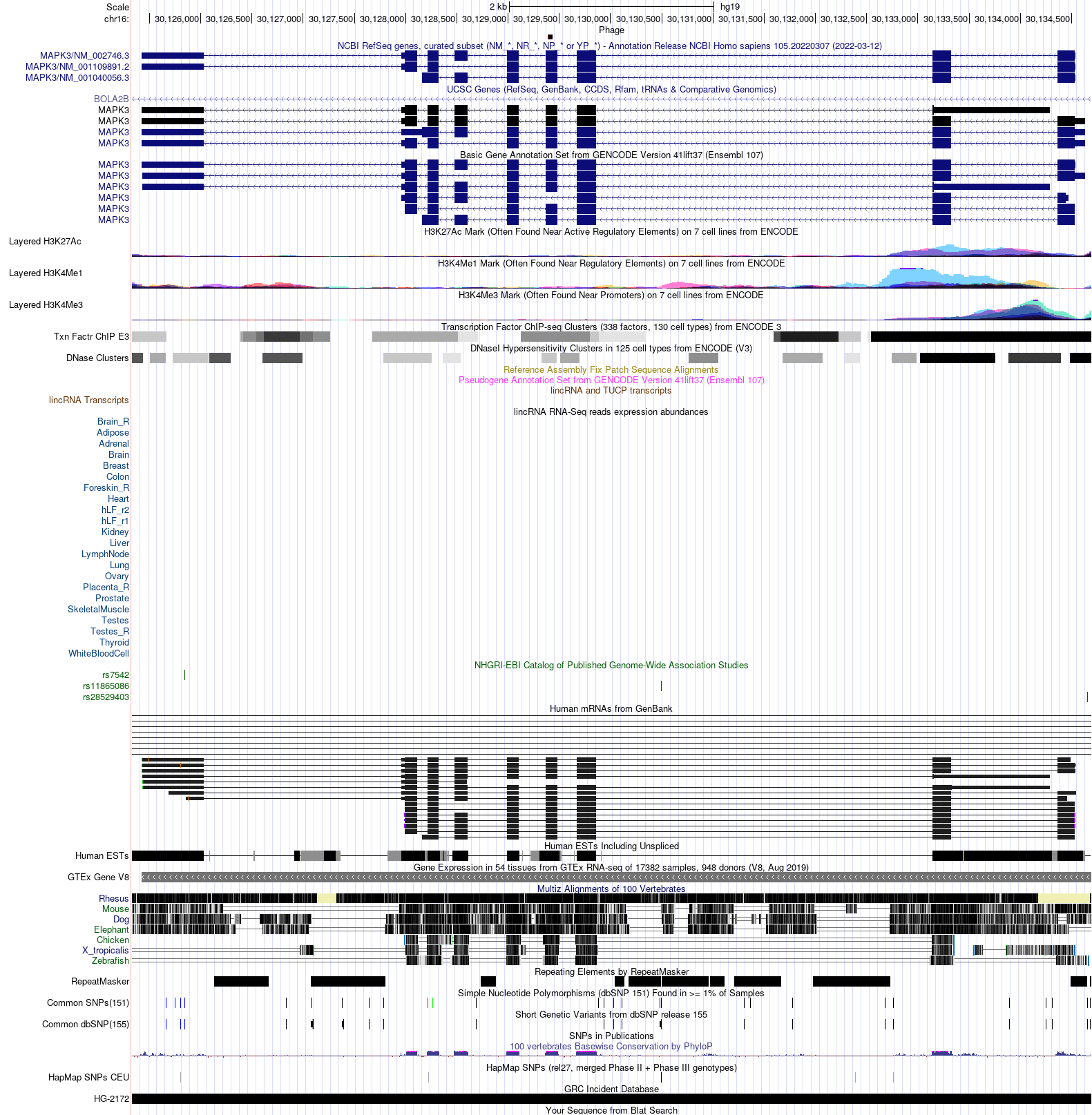


**UCSC Genome Browser results for *SPATA5.***


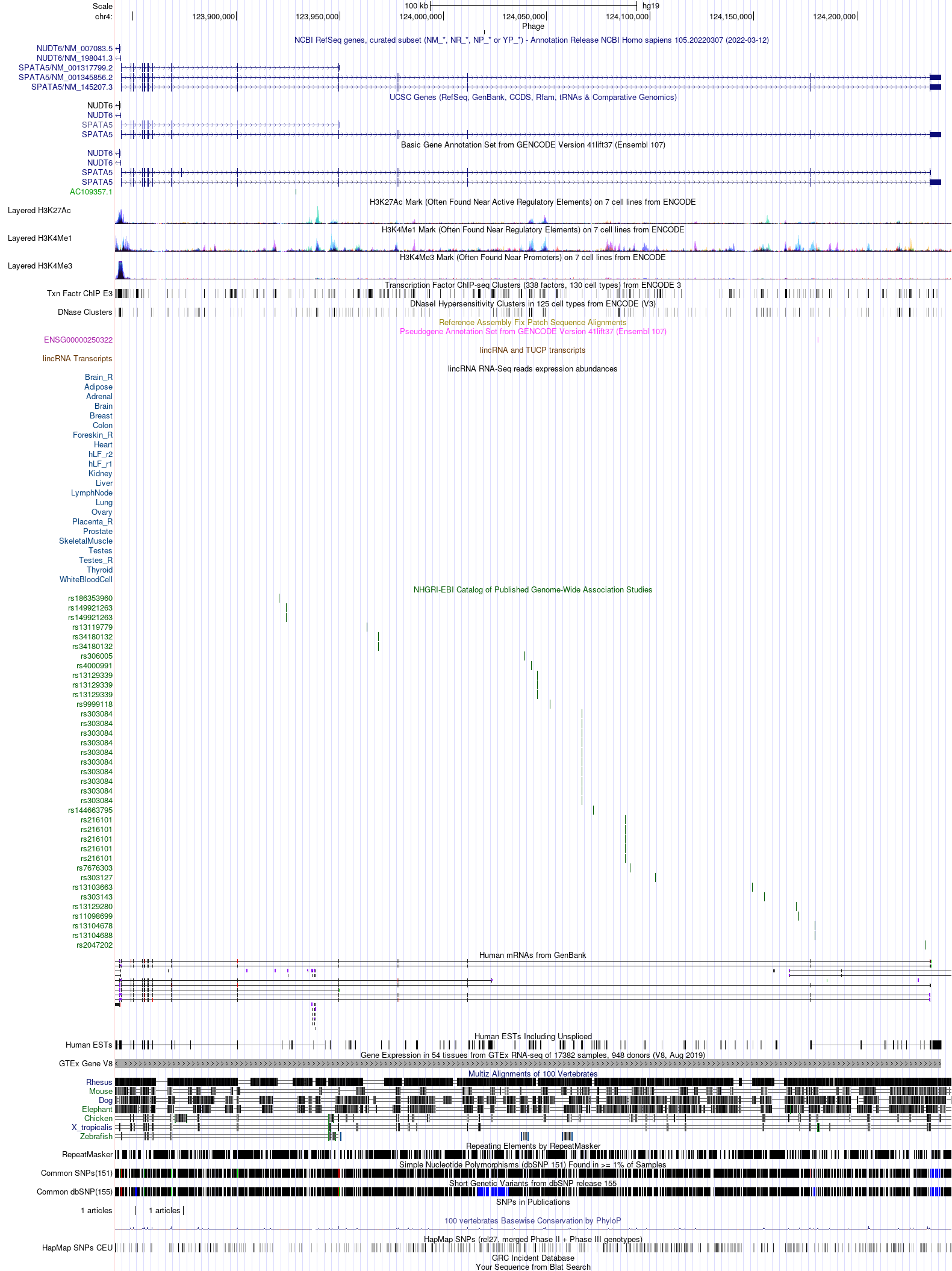


**UCSC Genome Browser results for *MFF.***

**
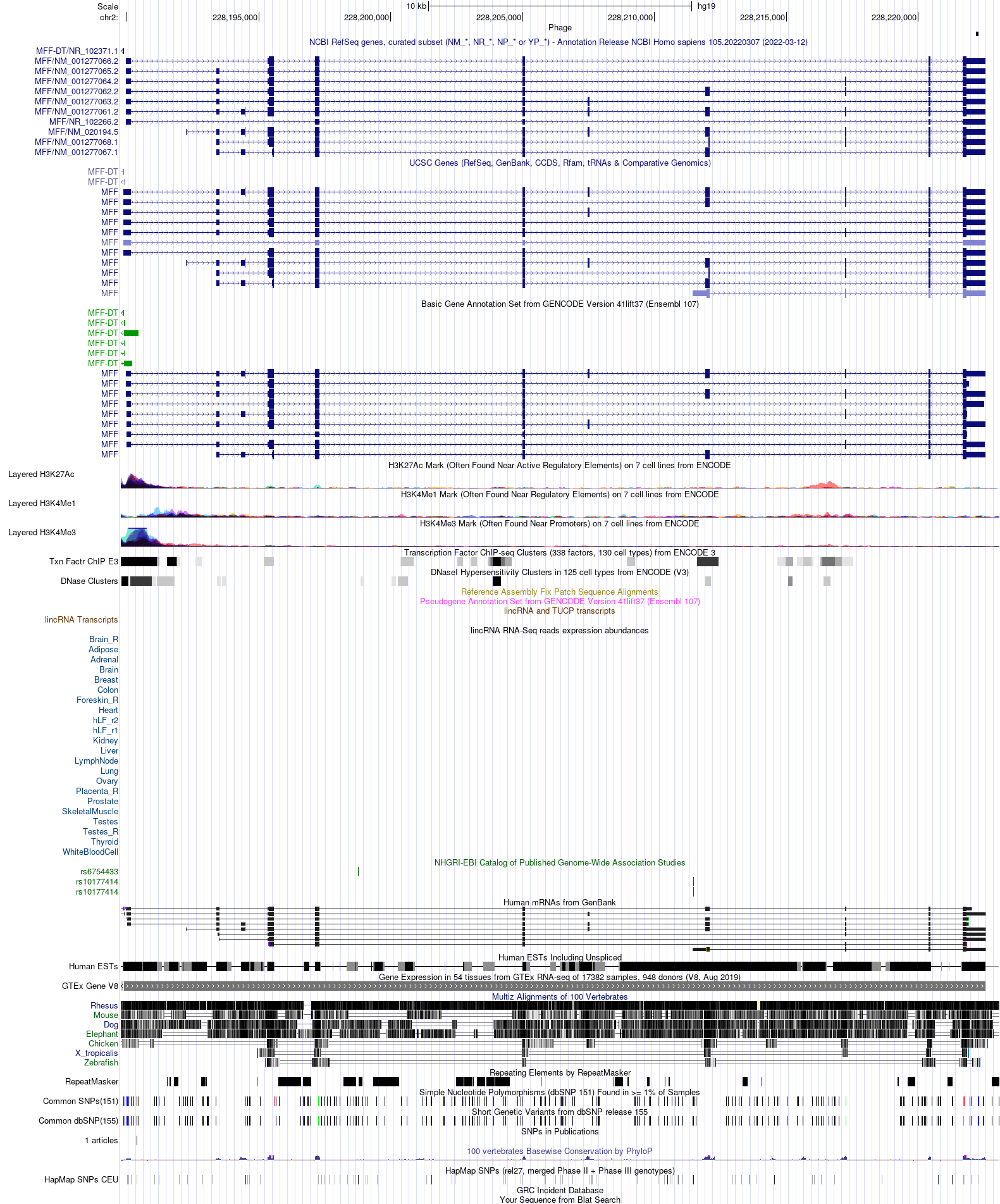
**

**UCSC Genome Browser results for *TSOAP1.***


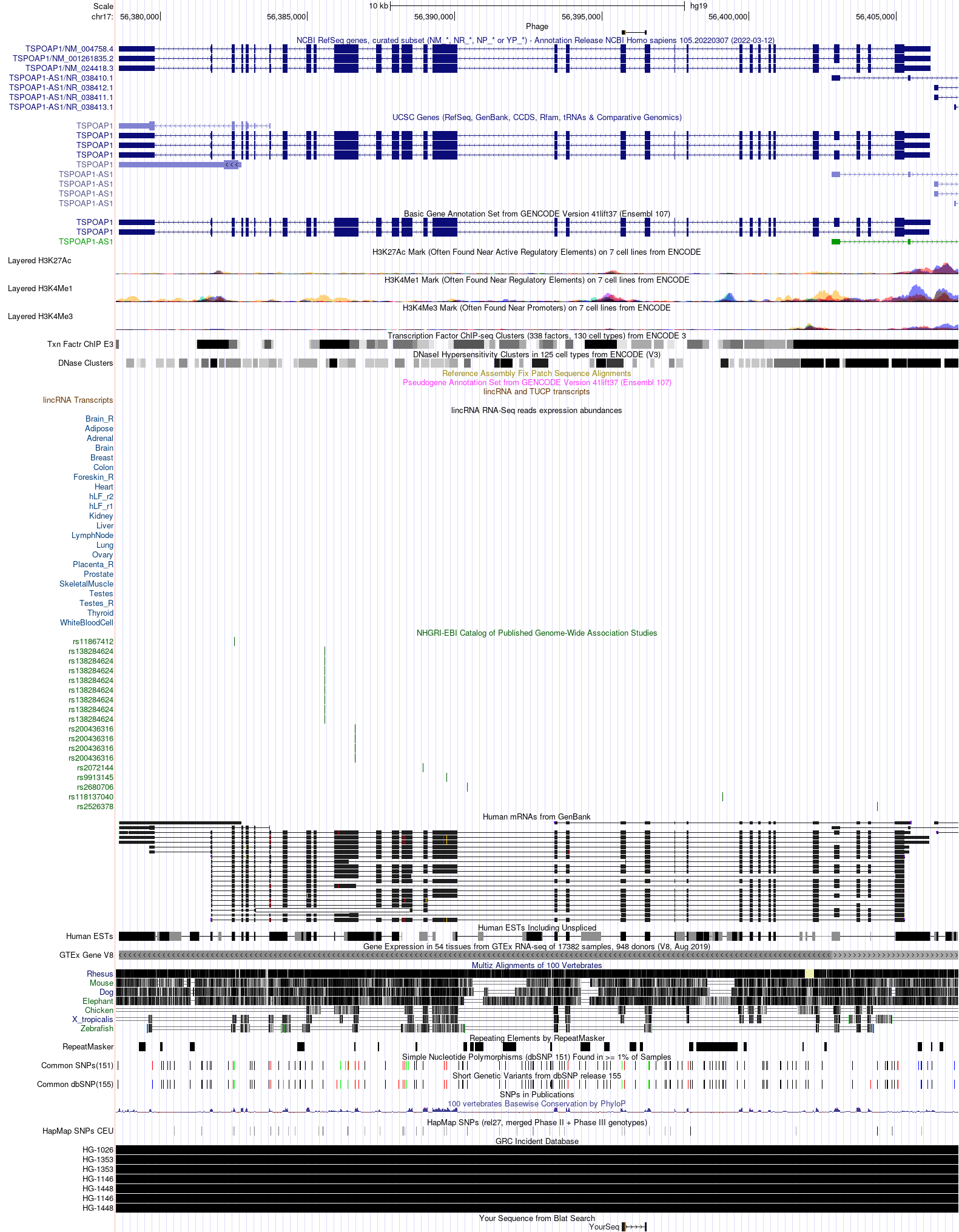


**UCSC Genome Browser results for *PHB2.***


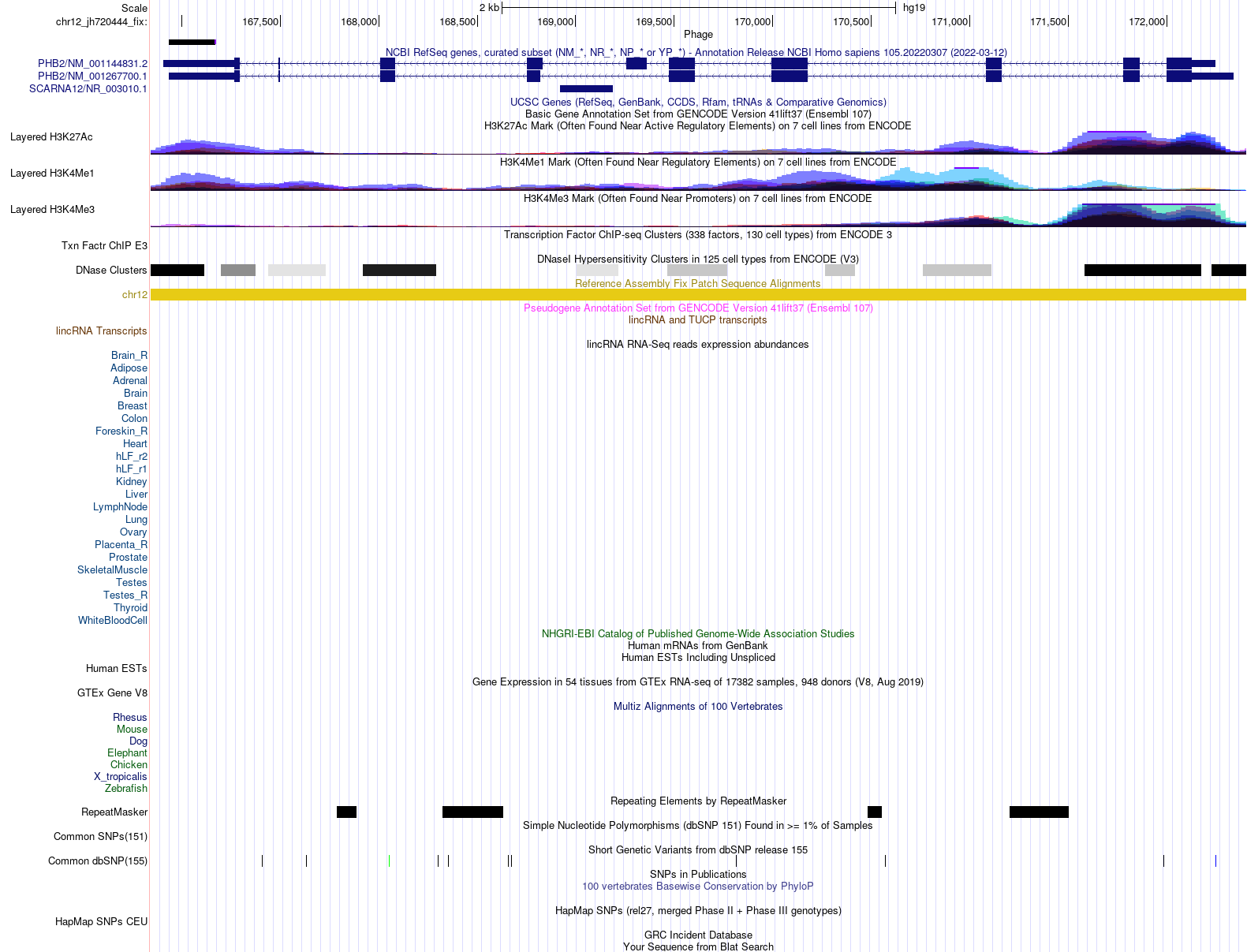


**UCSC Genome Browser results for *COA4.***


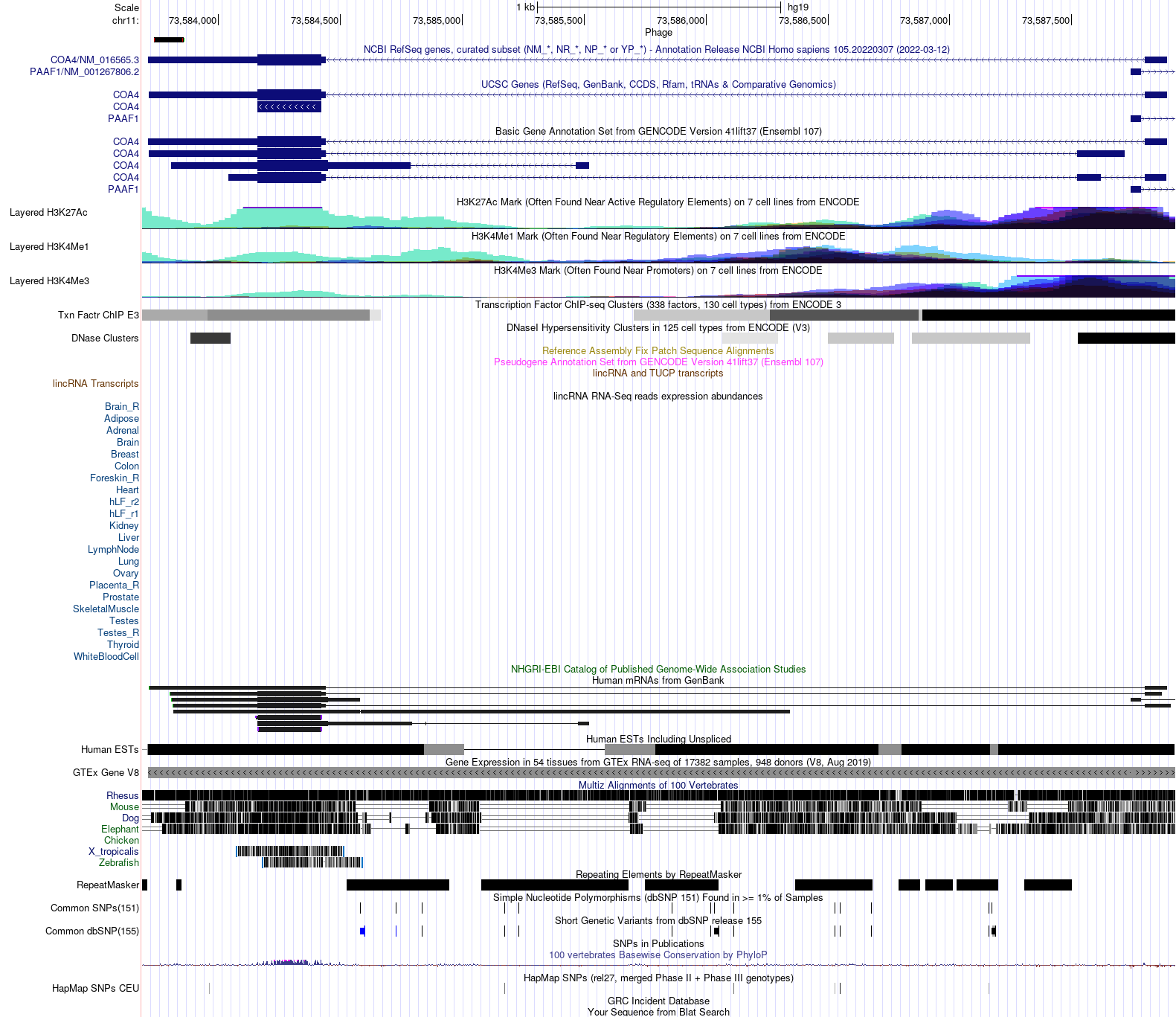


**UCSC Genome Browser results for *HAGH.***


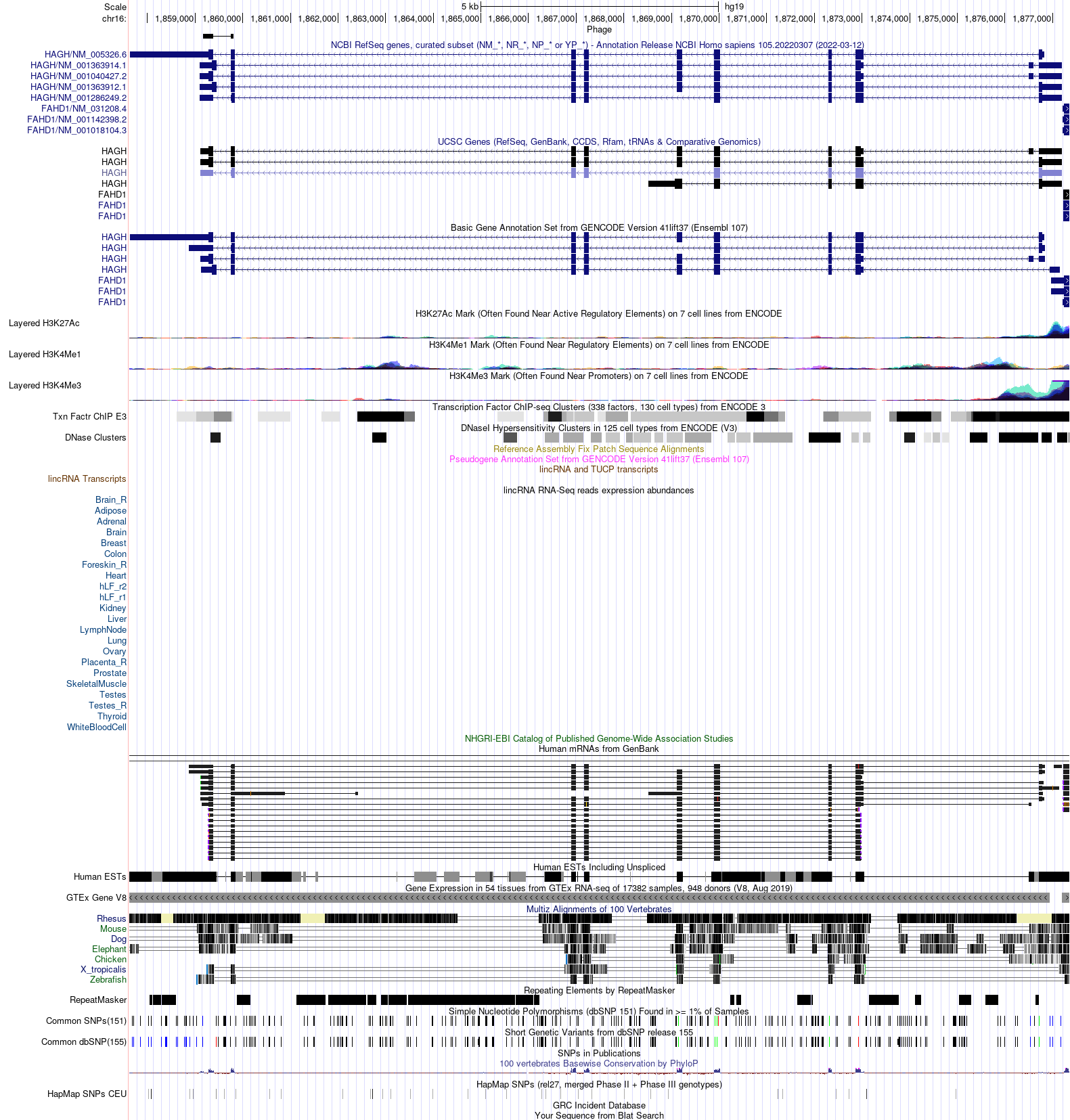


**UCSC Genome Browser results for *MNRR1* /** ***CHCHD2.***


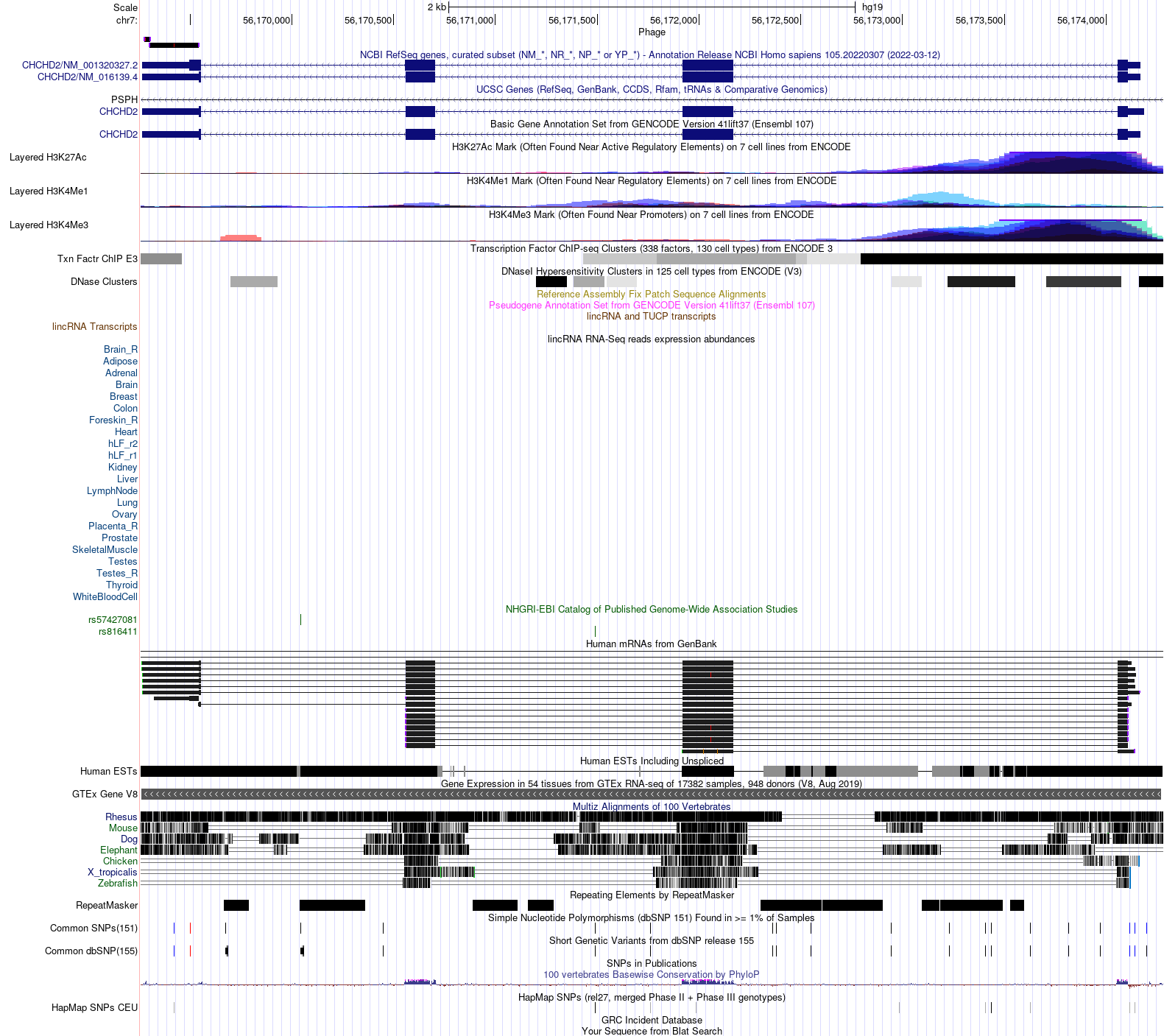


**UCSC Genome Browser results for *LINC02381.***


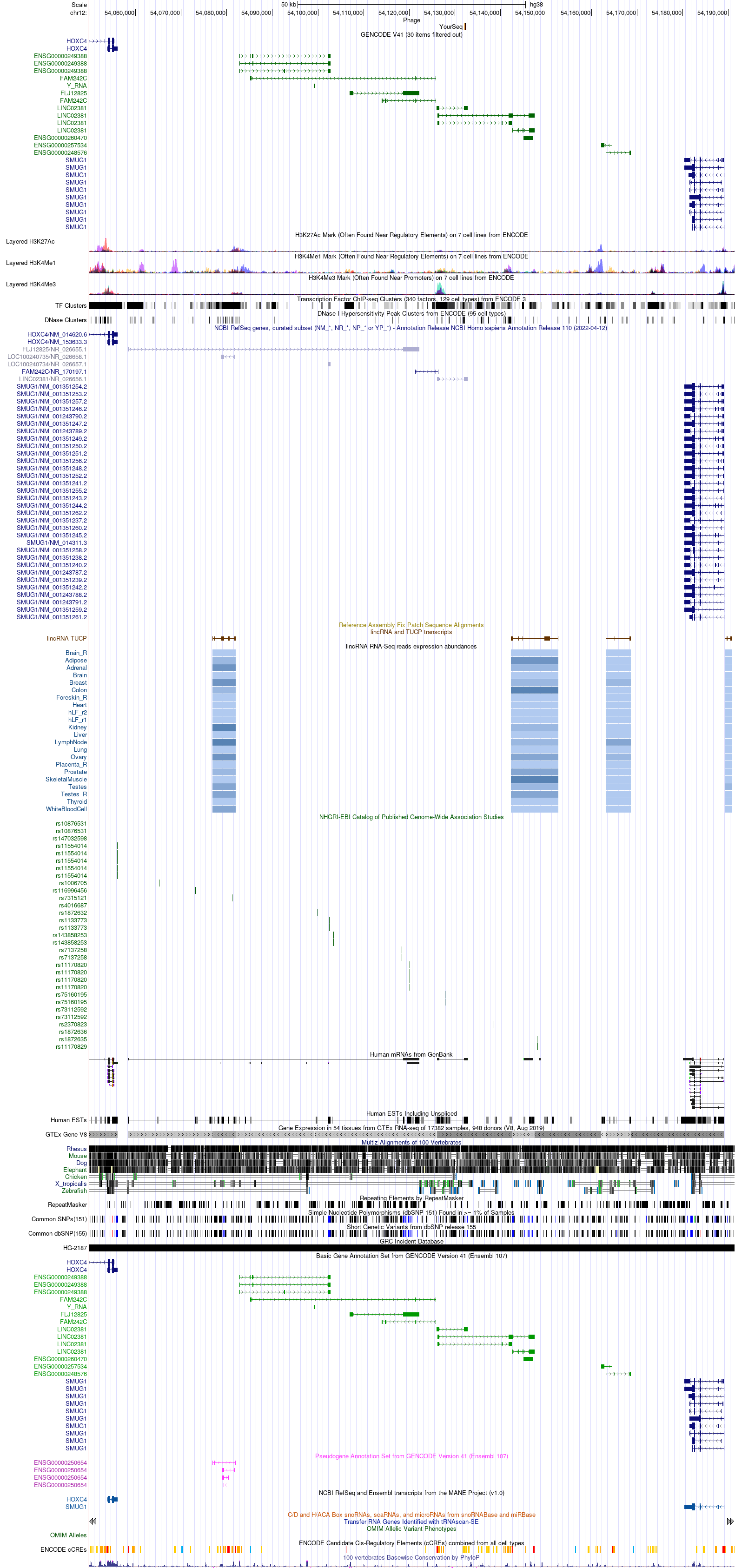


**UCSC Genome Browser results for intergenic neighbor of *CXCL13.***


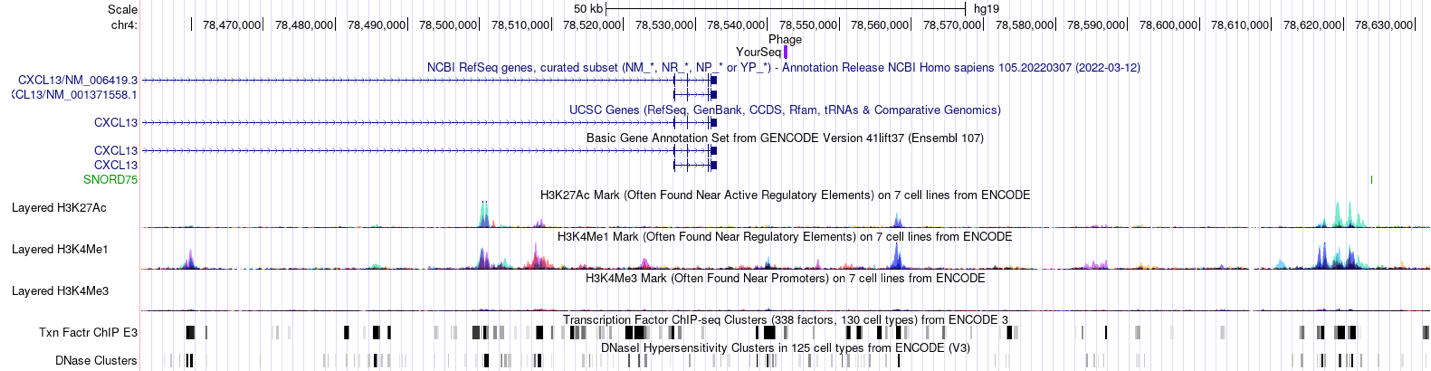


**UCSC Genome Browser results for intergenic/lncRNA neighbor of *ERCC4.***

**
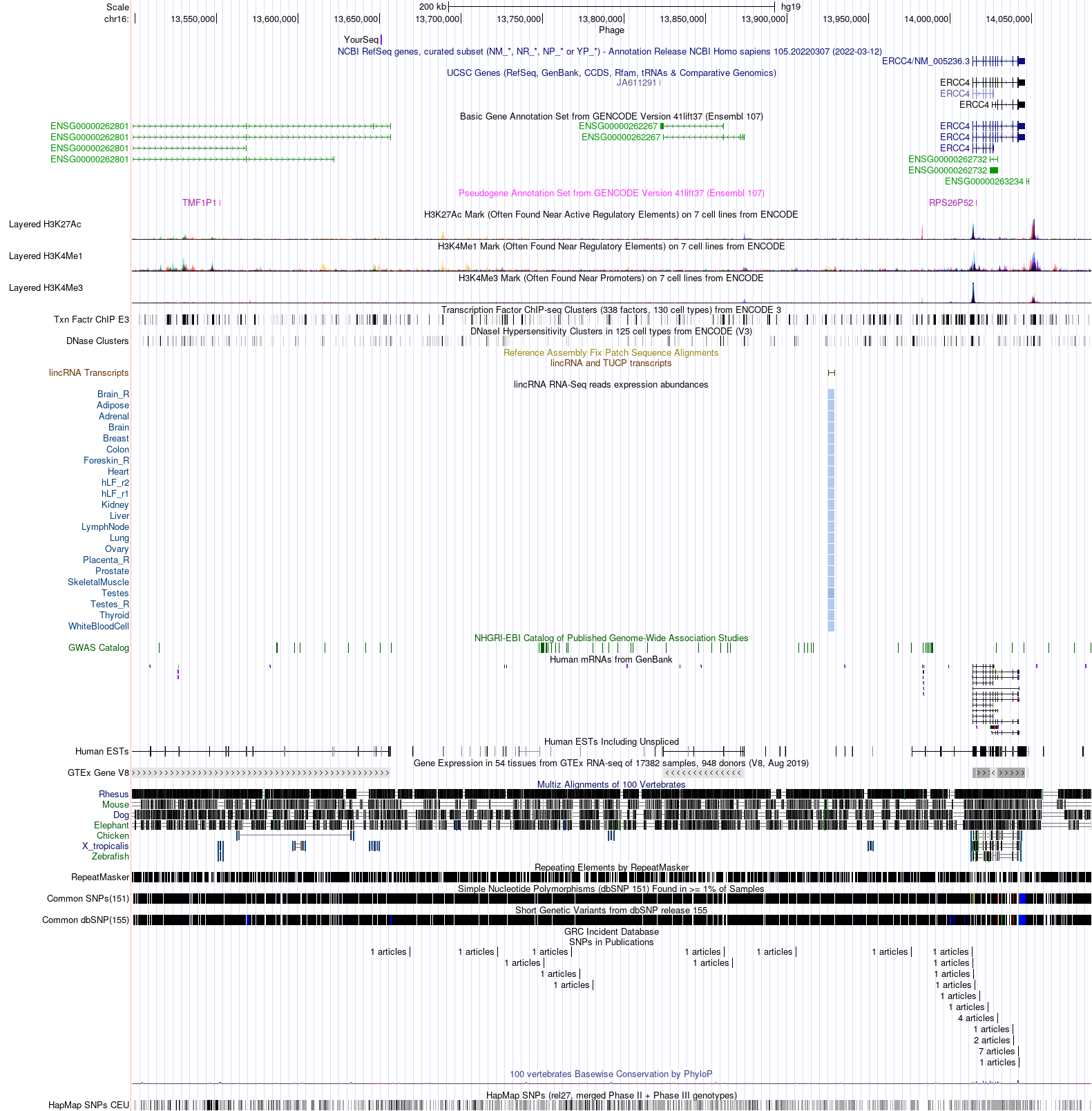
**

**UCSC Genome Browser results for intergenic neighbor of *SOX3.***

**
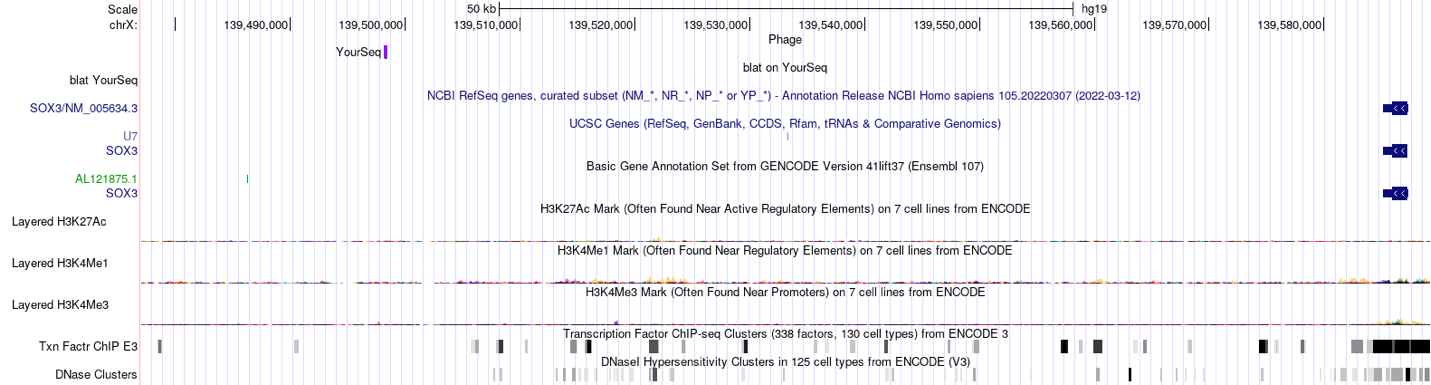
**

**UCSC Genome Browser results for the intergenic neighbor of *PCDH1.***

**
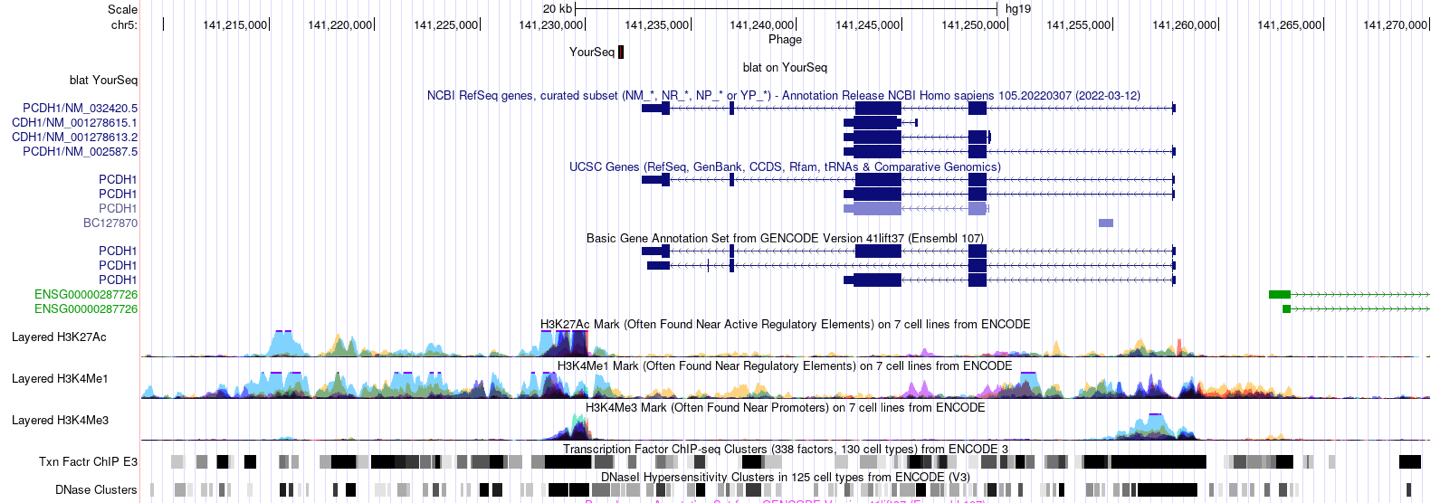
**

**UCSC Genome Browser results for the intergenic neighbor of *EDDM3B.***


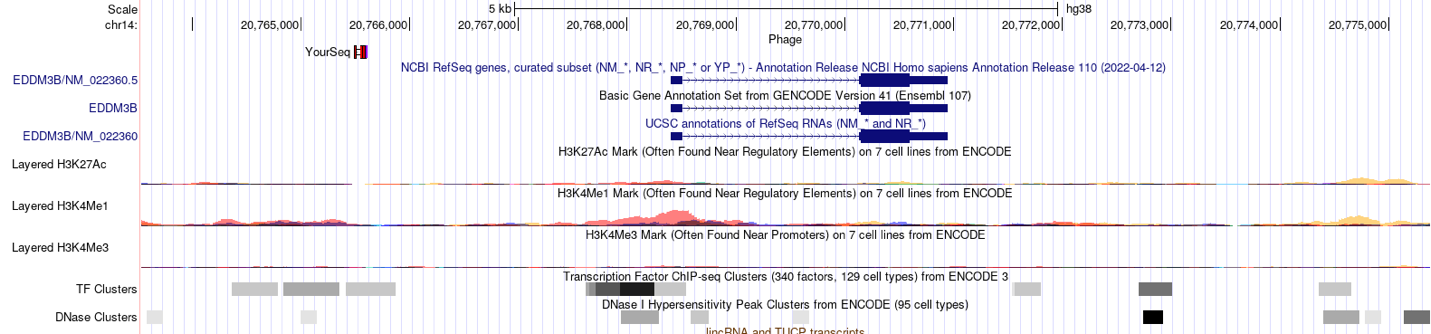


**UCSC Genome Browser results for intergenic neighbor of *GRB2.***

**
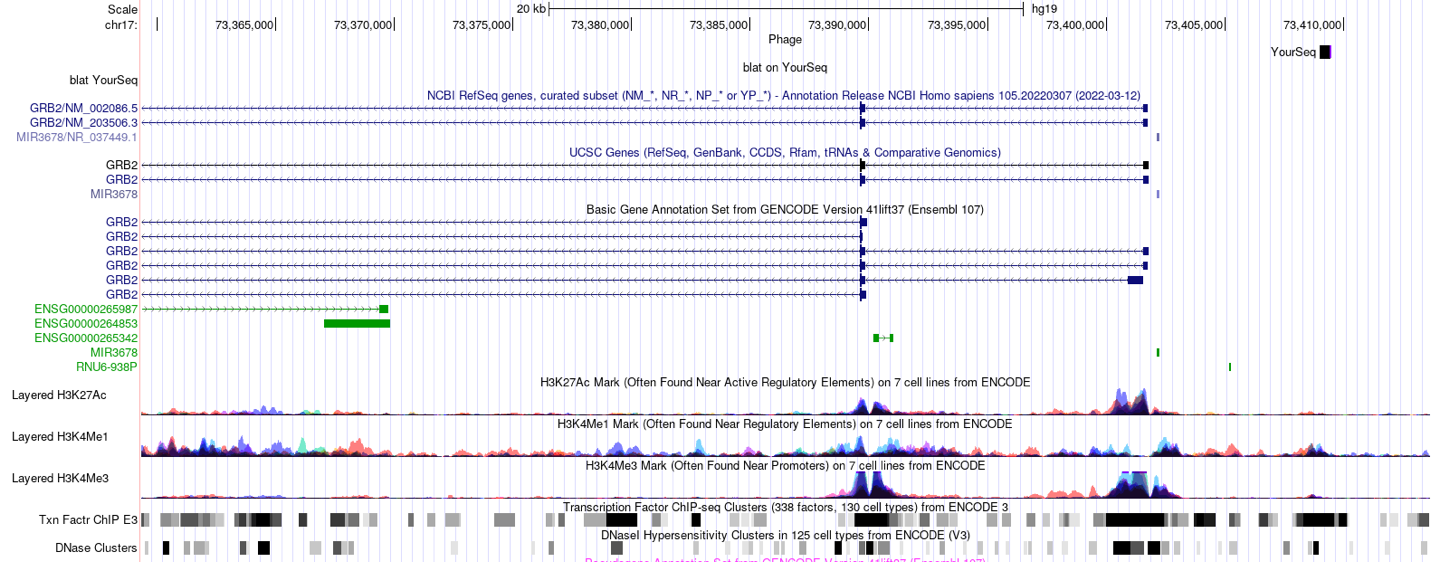
**

**4.Data Availability**

| Seq1 | *GAPDH* | OQ064295 |
| --- | --- | --- |
| Seq2 | *PKM2* | OQ158796 |
| Seq3 | *GSTP1* | OQ184077 |
| Seq4 | *COX7A2* | OQ214887 |
| Seq5 | *MAPK3* | JZ986783 |
| Seq6 | *SPATA5* | OQ286102 |
| Seq7 | *MFF* | JZ986781 |
| Seq8 | *TSPOAP1* | OQ320429 |
| Seq9 | *PHB2* | OQ268595 |
| Seq10 | *COA4* | JZ986784 |
| Seq11 | *HAGH* | OQ240960 |
| Seq12 | *LINC02381* | OQ240961 |
| Seq13 | *lncRNA* neighbor of *CXCL13* | OQ274133 |
| Seq14 | *lncRNA* neighbor of *ERCC4* | OQ274134 |
| Seq15 | *lncRNA* neighbor of *SOX3* | OQ274135 |
| Seq16 | *lncRNA* neighbor of *PCDH1* | OQ305204 |
| Seq17 | *lncRNA* neighbor of *EDDM3B* | JZ986782 |
| Seq18 | *lncRNA* neighbor of *GRB2* | OQ308776 |
